# Representativeness of the Natura 2000 network for preserving plant biodiversity in the European Union

**DOI:** 10.1111/cobi.70158

**Published:** 2025-10-12

**Authors:** Michele Di Musciano, Piero Zannini, Riccardo Testolin, Francesco Maria Sabatini, Diletta Santovito, Borja Jiménez‐Alfaro, Florian Jansen, Milan Chytrý, Lorenzo Ricci, Emiliano Agrillo, Fabio Attorre, Idoia Biurrun, Gianmaria Bonari, Hans Henrik Bruun, Luigi Cao Pinna, Andraž Čarni, Maria Laura Carranza, Roberto Cazzolla Gatti, Jürgen Dengler, Michele De Sanctis, Úna Fitzpatrick, Anna Rita Frattaroli, Emmanuel Garbolino, Stephan Hennekens, Ute Jandt, Jan Jansen, Zygmunt Kacki, Ilona Knollová, Jonathan Lenoir, Jesper Erenskjold Moeslund, Tomáš Peterka, Nataša Pipenbaher, Duccio Rocchini, Eszter Ruprecht, Solvita Rūsiņa, Urban Šilc, Sonja Škornik, Grzegorz Swacha, Milan Valachovič, Kiril Vassilev, Wolfgang Willner, Alessandro Chiarucci

**Affiliations:** ^1^ Department of Life, Health and Environmental Science University of L'Aquila L'Aquila Italy; ^2^ BIOME Lab Department of Biological Geological and Environmental Sciences Alma Mater Studiorum ‐ University of Bologna Bologna Italy; ^3^ Centro Interuniversitario per la Biodiversità Vegetale Big Data ‐ PLANT DATA Department of Biological Geological and Environmental Sciences Alma Mater Studiorum ‐ University of Bologna Bologna Italy; ^4^ LifeWatch Italy Lecce Italy; ^5^ Faculty of Forestry and Wood Sciences Czech University of Life Sciences Prague Prague Czech Republic; ^6^ Biodiversity Research Institute University of Oviedo Oviedo Spain; ^7^ Faculty of Agricultural and Environmental Sciences University of Rostock Rostock Germany; ^8^ Department of Botany and Zoology Faculty of Science Masaryk University Brno Czech Republic; ^9^ Italian Institute for Environmental Protection and Research – ISPRA Rome Italy; ^10^ Department of Environmental Biology Sapienza University of Rome Rome Italy; ^11^ Department of Plant Biology and Ecology Faculty of Science and Technology University of the Basque Country UPV/EHU Bilbao Spain; ^12^ Department of Life Sciences University of Siena Siena Italy; ^13^ Department of Biology University of Copenhagen Copenhagen Denmark; ^14^ Department of Science Roma Tre University Rome Italy; ^15^ School of Mathematics and Statistics University of Glasgow Glasgow UK; ^16^ Jovan Hadži Institute of Biology Research Centre of the Slovenian Academy of Sciences and Arts Ljubljana Slovenia; ^17^ School for Viticulture and Enology University of Nova Gorica Nova Gorica Slovenia; ^18^ EnvixLab, Dip. Bioscience and Territory University of Molise Pesche Italy; ^19^ NBFC, National Biodiversity Future Center Palermo Italy; ^20^ Vegetation Ecology Research Group Institut of Natural Resource Sciences (IUNR) ZHAW Zurich University of Applied Sciences Wädenswil Switzerland; ^21^ Bayreuth Center of Ecology and Environmental Research University of Bayreuth Bayreuth Germany; ^22^ National Biodiversity Data Centre Waterford Ireland; ^23^ MINES Paris PSL, ISIGE Fontainebleau France; ^24^ Alterra – Vegetation, Forest and Landscape Ecology Wageningen The Netherlands; ^25^ Geobotany and Botanical Garden Martin Luther University Halle‐Wittenberg Halle Germany; ^26^ German Centre for Integrative Biodiversity Research Halle‐Jena‐Leipzig Leipzig Germany; ^27^ Department of Ecology and Physiology, Faculty of Science Radboud University Nijmegen The Netherlands; ^28^ Botanical Garden University of Wrocław Wrocław Poland; ^29^ UMR CNRS 7058 Ecologie et Dynamique des Systèmes Anthropisés (EDYSAN) Université de Picardie Jules Verne Amiens France; ^30^ Department of Ecoscience Aarhus University Aarhus Denmark; ^31^ Faculty of Natural Sciences and Mathematics University of Maribor Maribor Slovenia; ^32^ Faculty of Environmental Sciences, Department of Spatial Sciences Czech University of Life Sciences Prague Prague Czech Republic; ^33^ Hungarian Department of Biology and Ecology Babeș‐Bolyai University Cluj Romania; ^34^ Faculty of Geography and Earth Sciences University of Latvia Riga Latvia; ^35^ Institute of Biology Research Centre of the Slovenian Academy of Sciences and Arts (ZRC SAZU) Ljubljana Slovenia; ^36^ Plant Science and Biodiversity Center Slovak Academy of Sciences Bratislava Slovakia; ^37^ Department of Plant and Fungal Diversity and Resources, Institute of Biodiversity and Ecosystem Research Bulgarian Academy of Sciences Sofia Bulgaria; ^38^ Department of Botany and Biodiversity Research University of Vienna Vienna Austria; ^39^ Vienna Institute for Nature Conservation & Analyses (VINCA) Vienna Austria

**Keywords:** area‐based conservation, gap analysis, Habitats Directive, Natura 2000, plant diversity, análisis de brechas, conservación basada en el área, Directiva Hábitats, diversidad vegetal, Natura 2000, 基于区域的保护, 空缺分析, 《栖息地指令》, Natura 2000, 植物多样性

## Abstract

The Natura 2000 (N2K) network of protected areas is one of the main tools for area‐based conservation in the European Union (EU), yet its role in preserving plant biodiversity requires better understanding. We examined data kept in the European Vegetation Archive from over 1.2 million vegetation plots and obtained over 14.2 million plant species occurrences. To test the N2K network's representativeness of plant species gamma diversity, we compared the number and percentage of native and conservation priority species in‐ and outside the N2K network throughout the EU and for individual countries, biogeographical regions, and combinations thereof. We then determined whether N2K sites hosted more species than sites outside the network with the species–area relationship. Overall, almost 90% of the native vascular plant species occurred at least once in the N2K network. Yet, significant variation exists across countries and biogeographical regions—from 0% of species in the Boreal region of Lithuania, to 98% in the Alpine region of Croatia—indicating that local N2K sites are not equally representative of the regional gamma diversity. Nonetheless, the N2K network contains more species than land outside the network when area is taken into account. The planned expansion of the N2K network, as mandated by the European Biodiversity Strategy for 2030, should prioritize areas with currently underrepresented elements of the EU vascular flora.

## Abstract (es)

Representatividad de la red Natura 2000 para la conservación de la diversidad vegetal en la Unión Europea

**Resumen:**

La red Natura 2000 (N2K) de áreas protegidas es una de las principales herramientas para la conservación basada en áreas de la Unión Europea (UE), pero su papel en la preservación de la biodiversidad vegetal requiere una mejor comprensión. Analizamos los datos conservados en el Archivo Europeo de Vegetación de más de 1.2 millones de parcelas de vegetación y obtuvimos más de 14.2 millones de ocurrencias de especies vegetales. Para comprobar la representatividad de la red N2K en cuanto a la diversidad gamma de especies vegetales, comparamos el número y el porcentaje de especies autóctonas y prioritarias para la conservación dentro y fuera de la red N2K en toda la UE y para cada país, región biogeográfica y combinaciones de ambas. A continuación, determinamos si los sitios N2K albergaban más especies que los sitios fuera de la red con la relación especie‐área. En general, casi el 90% de las especies de plantas vasculares autóctonas aparecieron al menos una vez en la red N2K. Sin embargo, existen variaciones significativas entre países y regiones biogeográficas, desde el 0% de las especies en la región boreal de Lituania hasta el 98% en la región alpina de Croacia, lo que indica que los sitios locales de la red N2K no representan de igual manera la diversidad gamma regional. No obstante, la red N2K contiene más especies que las zonas fuera de la red si se tiene en cuenta la superficie. La ampliación prevista de la red N2K, según lo dispuesto en la Estrategia Europea de Biodiversidad para 2030, debería dar prioridad a las zonas con elementos actualmente infrarrepresentados de la flora vascular de la UE.

## Natura 2000网络在保护欧盟植物生物多样性方面的代表性

**【摘要】** Natura 2000(N2K)保护区网络是欧盟基于区域的保护的主要工具之一, 但其在保护植物生物多样性方面的作用仍有待评估。本研究分析了“欧洲植被档案”数据库中保存的120多万个植被地块的数据, 获得了1,420多万条植物物种出现记录。为检验N2K网络在植物物种γ多样性方面的代表性, 我们比较了整个欧盟、欧盟的各个国家、生物地理区域及其组合中N2K网络内外的原生物种和保护物种的数量和百分比。随后, 我们利用种面积关系确定了N2K位点内部是否比外部存在更多的物种。总体而言, 近90%的原生维管植物物种在N2K网络中至少出现过一次。然而, 不同国家和生物地理区域之间存在很大差异——从立陶宛北方地区的0%到克罗地亚阿尔卑斯地区的98%——这表明局部的N2K位点在区域γ多样性方面的代表性不同。尽管如此, 如果考虑到区域因素, N2K网络包含的物种仍多于网络外的地区。根据《2030年欧洲生物多样性战略》的要求, N2K网络的扩展计划应优先考虑目前在欧盟维管植物区系中代表性不足的地区。**【翻译:胡怡思;审校:聂永刚】**

## INTRODUCTION

Anthropogenic pressure on ecosystems is intensifying worldwide and causing a rapid redistribution and decline of biodiversity (IPBES, 2019; Lenoir et al., 2020; Pecl et al., 2017) and biotic homogenization; generalist species are favored (Staude et al., 2020). These rapid biodiversity changes call for increased land allocation to nature conservation (Tittensor et al., 2014). After the 1992 adoption of the Convention on Biological Diversity in Rio de Janeiro, the European Union (EU) settled one of the most ambitious plans for area‐based conservation of biodiversity worldwide, which led to the creation of the Natura 2000 (N2K) network of protected areas (hereafter N2K). This network was established through the combination of the Birds Directive (79/409/EEC ‐ 1979) and the Habitats Directive (92/43/EEC ‐ 1992), which hold all EU member states accountable for the protection of endangered animals, plants, and habitat types of community interest occurring in their territory (Ellwanger et al., 2018; Evans, 2012). Comprising more than 26,000 sites and covering 752,257 km^2^ (18.3% of the EU land area), the N2K has become the largest coordinated network of protected areas in the world (European Commission, 2022; Evans, 2012; Sundseth & Creed, 2008) and represents the main legal instrument for conserving biodiversity in the EU (Maxwell et al., 2020), although other types of protected areas also exist (Cazzolla Gatti et al., 2023; Maiorano et al., 2015). Yet, the effectiveness of the N2K network in protecting natural and seminatural habitats to prevent biodiversity loss is still debated (Concepción, 2021; Kati et al., 2015).

Among the different components of biodiversity, plants deserve particular attention because they account for the vast majority of Earth's biomass (Bar‐On et al., 2018), form the base of nearly all food webs, and are critical to ecosystem functioning and the delivery of essential ecosystem services, such as carbon sequestration, air and water purification, and food provision (Harrison et al., 2014; Lavorel, 2013; Pironon et al., 2024). Understanding the extent to which the N2K network is effective in protecting EU plant biodiversity is crucial for planning the network's future expansion required by the European Biodiversity Strategy, which establishes the target of protecting at least 30% of the EU land area by 2030 (European Commission, 2020).

Assessing the long‐term effectiveness of the N2K network requires comprehensive information regarding the biodiversity hosted within its boundaries. An effective conservation network is representative of the full variety of biodiversity features in a region, with individual protected sites contributing to maximizing this overall representation (Margules & Pressey, 2000). Determining the extent to which a protected area network encompasses the total species richness of a region (i.e., the gamma diversity) represents a measure of the network's representativeness (Kukkala & Moilanen, 2012; Smith & Theberge, 1986). Comprehensive information on the current representativeness of the N2K network may be used to identify missing or underrepresented species and recommend future improvements (Cunningham et al., 2021).

Several attempts have been made so far to measure the biodiversity in the N2K network. Previous studies quantified the amount of habitats hosted by the N2K network in specific biogeographical regions (e.g., Gameiro et al., 2020; Lukács et al., 2013). Comprehensive biogeographical analyses for entire taxonomic or functional groups exist but are limited to charismatic or endangered species, such as birds (495 species [Albuquerque et al., 2013]); selected threatened species (300 species [Trochet & Schmeller, 2013]); terrestrial vertebrates (842 species [Maiorano et al., 2015]); species of conservation priority (714 species [Gruber et al., 2012]); and 587 vascular plants (Ricci et al., 2024). As for vascular plants, extensive assessments are limited to individual regions (e.g., Chiarucci et al., 2008; Sperandii et al., 2020; Spiliopoulou et al., 2021), whereas continental‐extent studies considered only restricted subsets of the EU flora, for example, 464 species listed in Annex II of the Habitats Directive (Gruber et al., 2012), 61 species of conservation concern (Trochet & Schmeller, 2013), around 500 IUCN Red List and orchid species, and 300 common species (van der Sluis et al., 2016). These studies showed that, although the N2K network has avoided major gaps in species representation, inefficiencies persist. Substantial coverage gaps remain, especially for wide‐ranging and threatened species, and these vary across biogeographical regions due to differences in site selection strategies, land use, and human pressures (Ricci et al., 2024). Given the large number of terrestrial vascular plant species native to the EU (>15,000 [Essl et al., 2013]) and their functional and structural role in ecosystem functioning and trophic chains, the lack of an EU‐wide assessment of the N2K network representativeness in protecting the gamma diversity of vascular plants is a glaring knowledge gap.

The European Vegetation Archive (EVA) (Chytrý et al., 2016) is a major source of information on species composition of local plant communities across the continent. The EVA stores information from more than 2 million vegetation plots, which makes it the largest vegetation plot database available for Europe. The fast expansion of EVA over the last decade is now creating unprecedented opportunities for assessing the extent to which the N2K network protects vascular plants across European countries and biogeographical regions based on data from field‐based scientific surveys. The EVA has several problems shared with other biodiversity databases that are based on opportunistically collected data (Meyer et al., 2016), for example, geographical biases in data coverage and preferential sampling of rare and species‐rich communities (Chytry et al., 2016; Szymura et al., 2023). Therefore, any biodiversity assessment based on EVA needs to be complemented with other approaches rooted in ecological theory, such as the species–area relationship (Bruelheide et al., 2020; Lawton, 1999; Lomolino, 2000). The species–area relationship has been proposed as a useful approach to compare areas with uneven sampling effort to assist conservation planning by, for example, determining how much additional area is required to protect a given proportion of species richness (Desmet & Cowling, 2004) or assessing deviations from expected species richness in protected areas (Hoffmann et al., 2018). These techniques have already been applied to investigate continental (Hoffmann et al., 2018), national (Kallimanis et al., 2008), and regional (Chiarucci et al., 2012, 2008) patterns of species richness in protected areas.

We used data extracted from EVA to provide the first estimate of the number and proportion of vascular plant species and legally protected plant species (the latter here called priority species, namely those listed in the Habitats Directive Annexes II, IV, and V) that occur in the N2K network across EU countries and biogeographical regions. We sought to estimate the representativeness of the N2K network with respect to the gamma diversity of vascular plant species throughout the EU and in each biogeographical region, country, and their combinations; determine in which countries and biogeographical regions the protected area network underperforms compared with the EU average while controlling for sampling effort; and test for differences in the number of species found inside and outside the N2K network with species–area curves.

## METHODS

### Study area

Across the EU terrestrial area, N2K coverage varies nationally from 7.2% in Denmark to 37.8% in Slovenia (EEA, 2020). The coverage of the N2K network is even more heterogeneous across biogeographical regions, ranging from 8.2% of the Boreal region to 57.6% of the Black Sea region (EEA, 2019, 2020). Alpine (39.4%) and the Macaronesian (38.1%) regions are also well covered by the N2K network.

### Data gathering and harmonization

We retrieved 1,223,017 vegetation plots from EVA (accessed on 16 March 2021), sampled across the 27 EU countries. From this set of plots, we selected only those with information on location uncertainty up to 10 km. Less than 1% of plots had such high location uncertainty, which had a median value of 10 m. We excluded plots from human‐made habitats (V category according to EUNIS classification [Moss, 2008] as provided by EVA) and plots sampled before 1994 (i.e., the year the N2K network was formally established). The total number of vegetation plots considered was therefore reduced to 769,157, corresponding to 14.2 million occurrences of native vascular plant species. Most of the plots in EVA were assigned to their respective country and biogeographical region. If this information was missing, plot location was overlaid with the map of biogeographical regions of Europe (Cervellini et al., 2020) and national boundaries (South, 2011). Then, we classified plots as falling inside or outside the polygons of the N2K shapefile (EEA, 2018) by demarcating circular buffers around plot coordinates with a radius equal to plot location uncertainty. The plots were considered inside the N2K network when >50% of the buffer area overlapped a polygon of the N2K shapefile. Most of the plots had their buffer largely (i.e., >75%) inside or outside of the network; intermediate situations (50–75%) were relatively rare. After this classification, 6.1 million native vascular plant species occurrences were recorded inside the N2K polygons, and 8.1 million were recorded outside.

To harmonize species names, we used the tool by Jansen (2023) based on Dengler et al. (2012) and data in Euro+Med, which follows the Euro+Med taxonomy (Euro+Med, 2006). We also used Euro+Med data to discriminate between native and non‐native species based on the reported taxon status in different areas. We removed a few occurrences for which the taxonomic identity could not be resolved. Finally, we selected species of conservation priority based on the last European report (EEA, 2020) to run separate analyses on this subset.

### Data analyses

**FIGURE 1:**
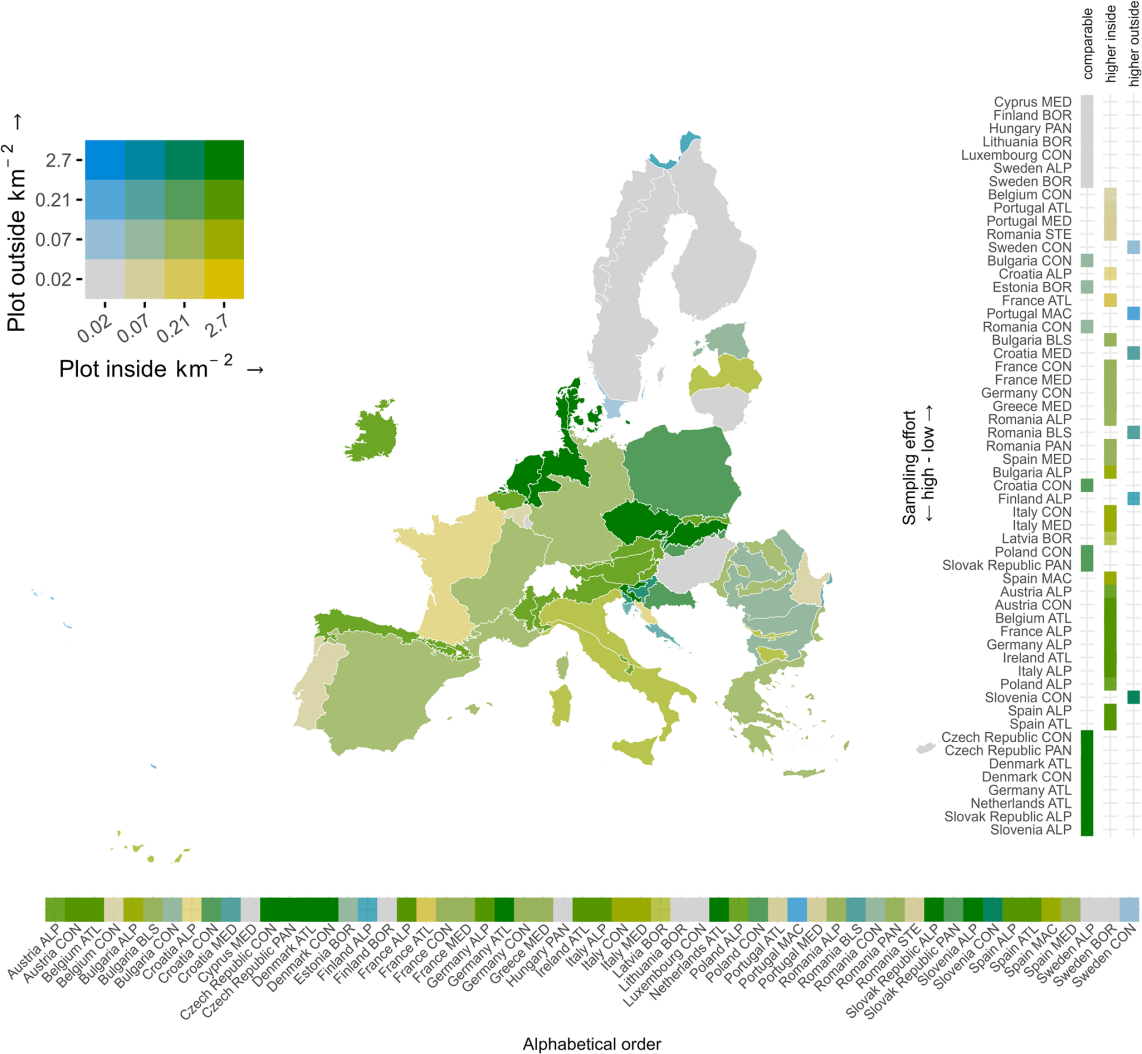
Vegetation plot density inside and outside the Natura 2000 network across combinations of countries and biogeographical regions (source European Vegetation Archive) (ALP, Alpine; ATL, Atlantic; BLS, Black Sea; BOR, Boreal; CON, continental; MAC, Macaronesian; MED, Mediterranean; PAN, Pannonian; STE, Steppic). Color classes are based on the quartiles of the distribution of plot density inside and outside the Natura 2000 network.

Species compositional diversity in an area can be observed at 3 distinct levels: alpha, beta, and gamma. Because our aim was to disentangle the role of protected areas in representing the gamma diversity at biogeographical or national scale, we focused on the total number of species beyond the scale of the single protected areas of the N2K network. Specifically, we calculated the cumulative number of native and priority species occurring in the entire set of selected vegetation plots, inside and outside the N2K network throughout the EU and in each biogeographical region, country, and their combinations. To avoid spurious results due to the incomplete area coverage of the EVA data, outputs related to combinations of biogeographical regions and countries that showed a strong bias in sampling effort (plots falling almost exclusively inside or outside the N2K network) were not reported. Specifically, we excluded combinations in which the sampling imbalance between plots inside and outside the network exceeded a factor of 10. This threshold was chosen as a trade‐off between retaining a sufficient number of regions and ensuring robust comparisons between protected and unprotected areas. This led to the exclusion of Malta from the study. To highlight differences in sampling effort among combinations of countries and biogeographical regions included in the study, we calculated their sampling density as the number of vegetation plots per square kilometer (Figure 1; Appendices S1 & S2).

For each country, biogeographical region, and their combinations, we also estimated the total number of native species with the Chao2 estimator inside and outside the N2K network and across the entire EU. This further step was performed to mitigate the effect of differences in sampling efforts across spatial units and provide an estimate of the overall gamma diversity found inside and outside the N2K network, disregarding species identities. The Chao2 estimator derives from the Cauchy–Schwarz inequality and returns a nonparametric estimation of the species richness of an assemblage based on species incidence (detection or nondetection) data in multiple sampling units. The results are nearly unbiased when very rare or infrequent species have approximately the same detection probabilities (Chao & Colwell, 2017). However, as with most of the other nonparametric estimators of species richness, Chao2 underestimates the total number of species in real communities and can be considered a lower bound estimator (e.g., Chiarucci et al., 2018; Xu et al., 2012). The Chao2 estimator was calculated with the iNext package 3.0.0 in R (Hsieh et al., 2016). A robustness check of our results was done comparing Chao2 with other common species richness estimators (i.e., the Michaelis–Menten and asymptotic regression model), which yielded equivalent results (Pearson correlation *r* = 0.98–0.99) (Appendix S3).

To evaluate the vascular plant species richness hosted in the N2K network, we calculated the proportion of native species found exclusively inside and outside the whole N2K network and the number of species shared between areas inside and outside of it in each country, biogeographical region, and their combinations. This proportion was calculated for observed and estimated (Chao2) species richness values. The proportion of estimated species richness inside the network for each country, biogeographical region, and their combinations was also related to the respective percentage of protected land surface.

We used a slightly different approach for priority species. We first compared the list of priority species found in the EVA dataset with those included in the European Environment Agency (EEA) report (EEA, 2021). We did this separately for each country, biogeographical region, and their combinations. We then calculated the proportion of priority species found exclusively inside the N2K network, exclusively outside it, and in both. To obtain a measure of dataset coverage, we identified missing priority species, that is, those listed in the last EEA report (EEA, 2020) but not found in the EVA dataset (Appendix S4), and calculated their proportion for each country, biogeographical region, and their combinations. We found some priority species in countries or biogeographical regions where they had not been reported in the last European report (Appendix S5). This finding could point to the presence of monitoring gaps in such regions that should be accounted for during the next monitoring rounds; species movement and range shifts; and, less likely, taxonomic misidentification. Although underreporting in official datasets has been suggested in previous studies (e.g., Lison et al., 2017), the uncertainty surrounding the reason for the presence of these species in the EVA dataset led us to exclude them from the main analysis to avoid misinterpretation. However, to assess the robustness of our results, we repeated the analyses including all priority species, regardless of their reporting status. We calculated the correlation coefficient across the proportions of species found exclusively inside or outside the N2K network, the proportion of species inside and outside, and the proportion of missing species. The outcomes were highly consistent, with a correlation coefficient of 0.95 between the 2 approaches. This result supported the reliability of the main findings.

We then tested how the proportion of native and priority species in each country or biogeographical region deviated from the overall EU pattern. To do so, we fitted generalized linear models (GLMs). We assumed a binomial distribution of errors and regressed the proportion of species in the N2K network against countries or biogeographical regions. Two separate models were run with countries or biogeographical regions as a covariate for native and priority species. Some countries were excluded from these analyses due to the low number of available vegetation plots inside the N2K network (Malta) or the absence of priority species in the plots sampled inside the network (Cyprus and Luxembourg). The density (number per square kilometer) of plots inside the N2K network for countries and biogeographical regions was included as a covariate to account for the different sampling efforts across countries and biogeographical regions. Given the nonlinear nature of the species–area relationship, we included sampling effort as a linear and quadratic predictor. We then selected the best‐fitting models based on the corrected Akaike's information criterion (AICc) and the explained deviance (*D* ^2^).

Finally, we examined whether N2K sites hosted a higher‐than‐expected number of species given their current spatial extent with the species–area relationship. We fitted separate curves to the estimated richness of native species inside and outside the N2K network with estimates for each combination of biogeographical region and country. To improve interpretability of the species–area relationship parameters, we rescaled the area variable by expressing it in units of 100 km^2^ prior to model fitting. This adjustment ensured model parameters reflected a spatial scale more aligned with the minimum size of the spatial units analyzed. This avoided extrapolating species richness to implausibly small areas (e.g., 1 km^2^), which are outside the observed range of our data. The curves were fitted with different models: power function, extended power function, and Kobayashi function. We then compared the different models and chose the one with the lowest value of the AICc and estimated the bootstrap confidence intervals of the selected model's parameters.

### Independent validation

As a robustness check and for independent validation, we compared the proportion of native species inside, shared, and outside N2K areas as obtained with the EVA dataset with the same metrics but with plant occurrence data from the Global Biodiversity Information Facility (GBIF). Georeferenced observations of vascular plants in EU countries were extracted from the GBIF database (www.gbif.org) on 18 September 2023 (GBIF, 2023). We only selected occurrences with location uncertainty up to 5 km. Because GBIF data are prone to a few common error types, we used the R package CoordinateCleaner (Zizka et al., 2019) to remove occurrences whose coordinates corresponded to the centroid of capitals, countries, GBIF headquarters, and known biodiversity institutions, as well as occurrences in oceans or records falling outside the study area despite being labeled as part of it. To be consistent with the time frame selected for the EVA dataset, we conducted this study excluding all occurrence records collected before 1994. We then followed the same procedures as for the main dataset originating from EVA. We calculated the proportion of native species found exclusively inside and outside the N2K network and the number of species shared between areas inside and outside the N2K network for each country, biogeographical region, and their combinations.

To further test the consistency of our results, we accounted for the effect of sampling bias by applying plot matching techniques. We identified the treatment group (i.e., plot inside N2K sites) that was closely matched to the control group (i.e., plot outside N2K sites) based on different environmental factors and spatial proximity. Specifically, we used propensity score matching, which is the most widely used technique (Geldmann et al., 2019). Based on previous studies (Andam et al., 2008; Feng et al., 2021; Geldmann et al., 2019; Joppa & Pfaff, 2009; Negret et al., 2020), our matching covariates were mean annual temperature, annual precipitation, temperature seasonality, precipitation seasonality, biogeographical region, country, and elevation. Matching was done with the MatchIt package (Ho et al., 2011) with the nearest neighbor method without replacement. The caliper (tolerance level for matching plots inside and outside the N2K network) was set to 0.25 SDs, meaning that the maximum allowable distance between matched pairs was constrained to fall within this threshold. This choice was guided by literature (Cuenca et al., 2016; Ricci et al., 2024) and justified empirically based on the SD of the estimated scores. This criterion is widely endorsed because it strikes a balance between minimizing selection bias and maintaining an adequate number of matched pairs, thereby improving the validity and efficiency of causal inference in matching techniques. The ratio parameter (the desired ratio of plots inside and outside the N2K network) was set to 1.

## RESULTS

### Native and priority species in the N2K network

**FIGURE 2:**
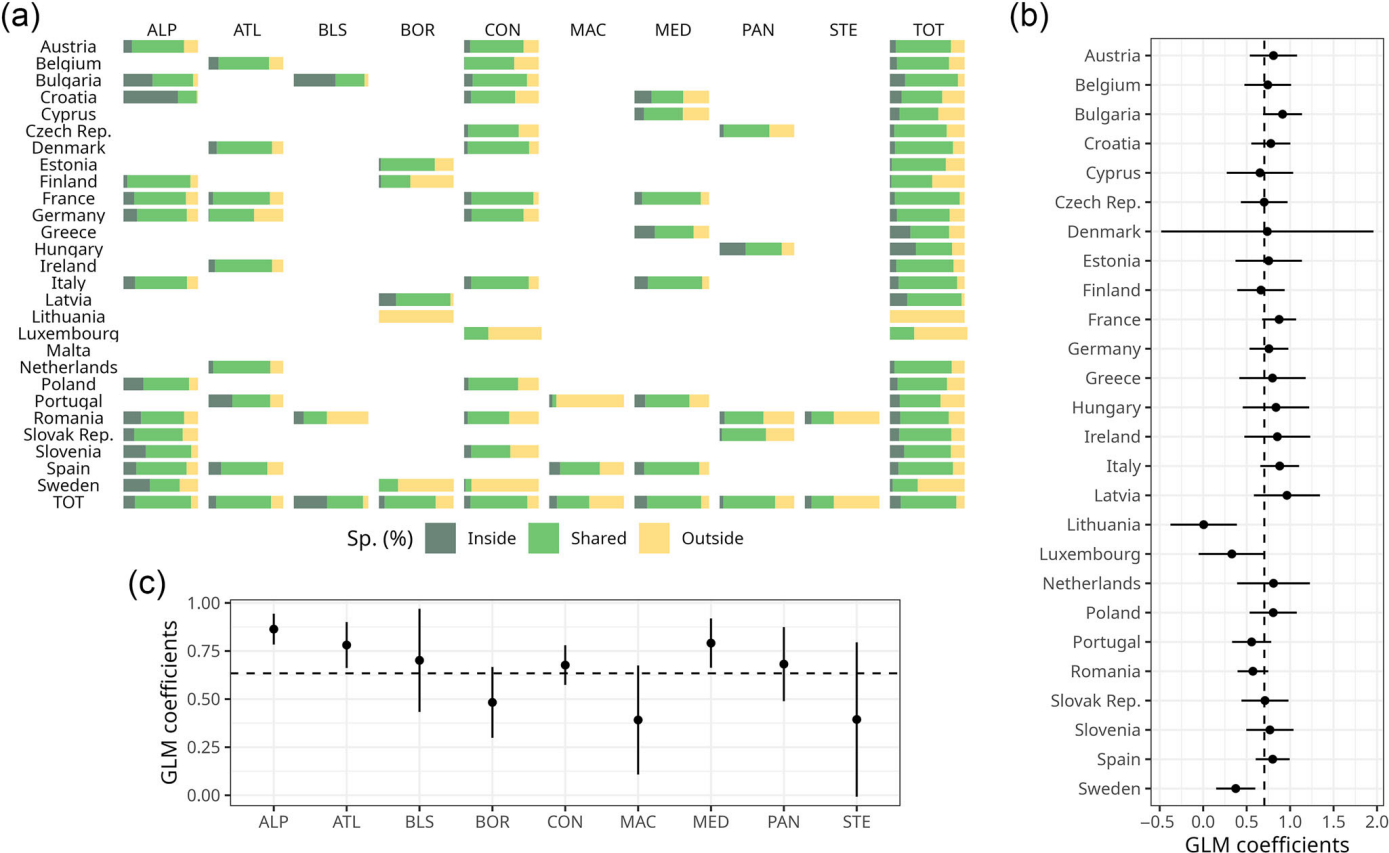
(a) Percentage of native species found exclusively inside, found exclusively outside, and shared between areas inside and outside the Natura 2000 network for each combination of country and biogeographical region of the EU and generalized linear model (GLM) coefficients of the percentage of species found in the network by (b) country and (c) biogeographical regions accounting for sampling effort (bars, 95% confidence interval; dots, mean of GLM coefficients; dashed lines, average pattern in Europe; ALP, Alpine; ATL, Atlantic; BLS, Black Sea; BOR, boreal; CON, continental; MAC, Macaronesian; MED, Mediterranean; PAN, Pannonian; STE, Steppic; TOT, total).

**FIGURE 3:**
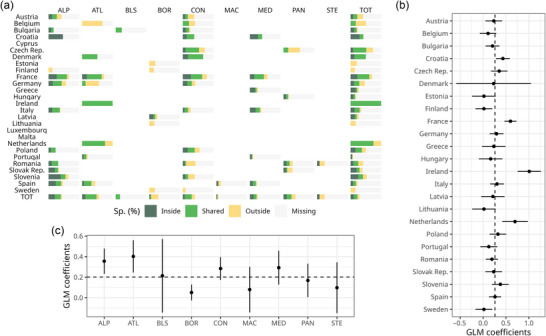
Percentage of (a) priority species found exclusively inside, found exclusively outside, and shared between areas inside and outside the Natura 2000 network compared with the total percentage reported for each combination of country and biogeographical region of the EU and the percentage of missing species and generalized linear model (GLM) coefficients of the proportion of priority species found inside the network by (b) country and (c) biogeographical regions accounting for sampling effort (black bars, 95% confidence interval; dots, mean of GLM coefficients; dashed lines, average pattern in Europe; ALP, Alpine; ATL, Atlantic; BLS, Black Sea; BOR, boreal; CON, continental; MAC, Macaronesian; MED, Mediterranean; PAN, Pannonian; STE, Steppic; TOT, total).

The overall number of native species recorded in the dataset for the entire EU was 9252, with 8186 (89%) and 7877 (85%) species recorded inside and outside the N2K network, respectively. As many as 6811 species were found inside and outside N2K polygons (74% of the total recorded native species), 1375 (15%) were exclusively found inside the N2K network, and 1066 (12%) were found exclusively outside. When estimating the species richness with Chao2, results were similar, with 89% of species inside and 86% outside the N2K network (Figure 2a). The overall number of priority species recorded across the EU was 239 (38% of the total Habitats Directive plant species), with 182 (30%) and 168 (27%) species recorded inside and outside the N2K network, respectively (Figure 3a; Appendix S6). Of these, 111 priority species were shared, representing 18.1% of the total number of the priority species of the EU; 71 (12%) priority species were only found in the N2K network.

### Native species in the N2K network across countries and biogeographical regions

Countries other than Cyprus, Finland, Lithuania, Luxembourg, Portugal, Romania, and Sweden had a proportion of native vascular plant species occurring in the N2K network (also considering shared species) above 70%. In most cases (except for Hungary), >50% of the species were shared between areas located inside and outside N2K polygons. Across the EU countries, the proportion of species exclusively found in N2K polygons ranged from 0% (Luxembourg) to 36% (Hungary) (Figure 2a; Appendix S7). After accounting for plot density, Bulgaria, France, Italy, and Latvia had significantly higher proportions of species recorded in the N2K network than the average (Figure 2b). Lithuania and Sweden, conversely, hosted significantly lower proportions of species in the N2K than the EU average. Adding plot density as a quadratic term to the model was not supported by the data because the quadratic term was never significant and did not increase the explained deviance or substantially change the AICc.

Concerning biogeographical regions, the proportion of estimated native vascular plant species recorded in the N2K network (also considering shared species) ranged from 38.8% (Steppic) to 93% (Black Sea). The Alpine, Mediterranean, and Black Sea regions hosted the highest proportion (∼90%) of estimated native species in the N2K network. The biogeographical region with the second lowest proportion of estimated species occurring in the N2K network was the Macaronesian (54%). Variable proportions of species were exclusive to the N2K network across biogeographical regions (average of 14%) (Figure 2a; Appendix S7). When accounting for plot density in each unit, biogeographical regions with a significantly higher proportion of species recorded in N2K sites were the Alpine, Atlantic, and Mediterranean (Figure 2c).

The proportion of native species in the N2K network (also considering shared species) for each combination of biogeographical region and country varied from 0% (Boreal region of Lithuania) to 98% (Alpine regions of Croatia). Most combinations of biogeographical region and country exhibited proportions of native species in the N2K network of >80%. Other combinations with a low proportion of native species in the N2K network were the Macaronesian region of Portugal (9%) and the Continental region of Sweden (10%) (Figure 2a; Appendix S7). We observed a positive relationship between the proportion of estimated species richness for each country, biogeographical region, and their combinations in the N2K network and the respective percentage of protected land surface (Appendices S7 & S8).

Use of the GBIF dataset revealed a strong correlation with the results obtained with the EVA dataset (Pearson correlation *r* > 0.80; Appendix S3). Analyses performed on the subset of environmentally matched plots also showed very similar results (Appendix S3), suggesting the dataset was environmentally balanced even before applying propensity score matching. Indeed, >75% of the plots were retained after the matching.

### Priority species in the N2K network across countries and biogeographical regions

Countries with the highest proportion of priority species occurring in the N2K network (also considering shared species) were Ireland (100%), the Netherlands (75%), and France (61%). Countries with no priority species found inside N2K polygons were Sweden, Estonia, Finland, and Lithuania, and Portugal and Hungary had relatively low proportions (6% and 15%, respectively). Luxembourg and Cyprus did not have any records of priority species in the dataset (Figure 3a,b; Appendices S6 & S7).

Biogeographical regions with the highest proportion of priority species occurring in the N2K network (also considering shared species) were the Atlantic (47%) and Alpine (40%), as opposed to the Macaronesia (3%), Boreal (6%), and Steppic (8%), where this proportion was the lowest (Figure 3a; Appendix S6) (Appendix S9 contains a version of the Figure 3a graph that excludes missing species). After accounting for the density of plots, the only regions where the proportion of priority species in the N2K network was higher than the EU average were the Alpine and Atlantic regions (Figure 3c; Appendix S6).

As for the number of priority species contained in the N2K network (also considering shared species), for each combination of biogeographical region and country, relatively high values were observed in the continental regions of Denmark (67%) and France (63%), in the Alpine regions of France and Slovenia (60% and 55%, respectively), and in the Atlantic region of France (64%). Combinations of biogeographical region and country with the lowest proportion of priority species in the N2K network were the Macaronesian region of Spain (7%), the Mediterranean region of Portugal (8%), and the Steppic region of Romania (8%). Several combinations had zero records of priority species in the N2K network, namely the Macaronesian region of Portugal, the Black Sea region of Romania, and the Atlantic region of Belgium (Figure 3a; Appendix S7).

### Species–area relationships

**FIGURE 4:**
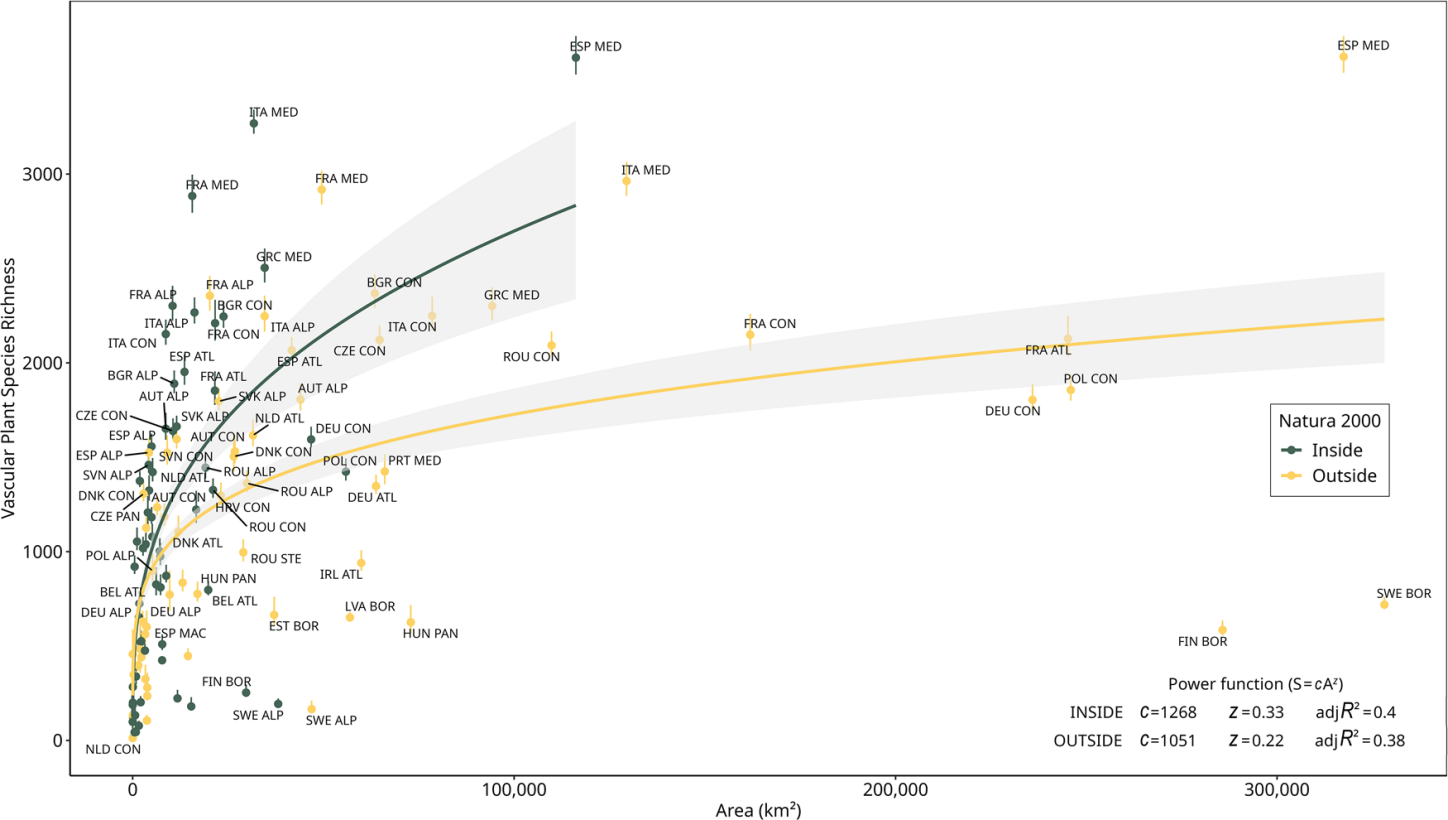
Species–area relationship for the total number of native species recorded inside and outside the Natura 2000 network for each combination of country and biogeographical region (dots, estimated species richness values for each combination of country and biogeographical region; bars, 95% confidence interval of the richness estimates; solid lines, species–area curves fitted with the power function; shaded areas, 95% bootstrap confidence intervals; ALP, Alpine; ATL, Atlantic; BLS, Black Sea; BOR, Boreal; CON, continental; MAC, Macaronesian; MED, Mediterranean; PAN, Pannonian; STE, Steppic).

The tested models exhibited negligible differences in AICc (power = 954; extended power = 956; Kobayashi = 955). Therefore, we fitted the species–area curves among combinations of countries and biogeographical regions with the power function because it was the easiest to interpret in terms of parameters and because it is widely used. Significant differences were observed between the scaling exponent *z* of the species–area curve fitted with the estimated richness of native species in the N2K network (*z* = 0.33) and *z* of the curve fitted with the richness outside the network (*z* = 0.22) (nonoverlapping 95% bootstrap confidence intervals). The species richness constant, *c*, representing the mean number of species in an area of 100 km^2^, also showed a higher value for the curve fitted with the estimated richness in the N2K network (*c* = 1268) than the one fitted with the richness outside of it (*c* = 1051) (Figure 4).

## DISCUSSION

It is surprising that 30 years after the enactment of the Habitats Directive and about 20 years after the actual establishment of the N2K network, an assessment at the extent of the EU for plant diversity hosted in the N2K network was still lacking. This was likely due to the difficulties in obtaining standardized biodiversity data at fine spatial grain at a continental extent. The gap has been partly filled in by the large amount of vegetation plot data collected in the field and collated in the EVA database (Chytrý et al., 2016), which currently encompasses roughly half of the overall European vascular plant species richness. Our results provided the first estimate of the representativeness of the N2K network with respect to the gamma diversity of vascular plants throughout the EU and in individual countries, biogeographical regions, and their combinations by including all native plant species and those of priority conservation interest according to the Habitats Directive.

Despite covering only one fifth of the EU area and hosting a low number of plant occurrences (6.1 vs. 8.1 million occurrences, as per the data used in this study), the N2K network appears to contain a significantly higher plant diversity than areas outside the network. This greater diversity inside the N2K network consists of more native species—even when taking area into account—and more priority species. Specifically, we found that 89% of vascular plant species in the EU had at least one occurrence in the N2K network, and only 12% of estimated species richness was found exclusively outside. This result is consistent with Ricci et al. (2024), who quantified the alpha, beta, and gamma diversity of 1769 priority species from EU's Birds and Habitats Directives and found higher diversity inside the N2K network than outside. These findings support the concept of representativeness of the N2K network for the gamma diversity of plant species in the EU. However, ours is only one of the many interpretations of the concept of representativeness, the definition of which remains unclear (Kukkala & Moilanen, 2012). We defined representativeness based on the number of species included at least once in the N2K network (Ferrier & Wintle, 2009). Although we found good coverage of plant diversity in the N2K, our results do not imply that all these species are adequately protected or that individual N2K sites are equally representative of local species richness. Indeed, our analyses are based on the proportion of gamma diversity—not the species distribution—included in the N2K. Nonetheless, our results reflect the state of the art 30 years after the adoption of the Habitats Directive, highlighting the progress made and the challenges that remain in achieving comprehensive species protection. The representativeness of the N2K network with respect to species ranges is required to further determine its effectiveness.

When taking area into account, the N2K network hosted more species than land outside the network. This finding might be related to the effectiveness of the selection of the N2K sites. Despite sometimes failing to protect target habitats and species due to poor planning (McKenna et al., 2014), the N2K network still covers a wide variety of natural and seminatural habitats (Chiarucci et al., 2012; Evans, 2012). Moreover, the greater number of species found in the network while accounting for area may also reflect the effectiveness of continuous EU investments in nature conservation (European Commission, 2022; Opermanis et al., 2012). Yet, areas inside the N2K network might have been sampled more intensively than the areas outside because the EU dedicates specific funding to monitor the N2K network and biodiversity‐rich areas (Sánchez‐Fernández et al., 2018), and these areas are generally of greater interest for plant ecologists. Although we cannot completely exclude that this is the case, the fact that our results were consistent when accounting for sampling intensity and when comparing the results with an alternative data source (GBIF) suggests they are robust and not just the result of sampling artefacts.

The magnitude of the positive association between N2K areas and plant species richness varied greatly across countries and biogeographical regions. Although these results might be due to insufficient sampling intensity in some areas (which seems likely for Sweden), they do provide some indications that N2K network might be poorly representative of the gamma diversity of vascular plants in some regions. Part of the variability may be due to different interpretations and implementation of the Habitats and Birds Directives across member states, at least within the boundaries set by the verdicts of the European Court of Justice. This may be due to the variable percentage of land and sea area set aside and to poor planning and site selection strategy, which may ultimately stem from differences in levels of conservation ambition among countries (Davis et al., 2014). For example, the European Commission started an infringement process in February 2024 for Portugal's failure to comply with previous judgments on the obligation for protection of 61 Sites of Community Importance under the Habitats Directive (Press Corner of the European Commission, 2024).

Less visible, but no less important, might be the differences across countries in the actual level of protection and enforcement of the N2K prescriptions, which has been observed to vary in the case of strictly protected areas (Cazzolla Gatti et al., 2023). In Denmark, for example, plantation forestry is allowed inside N2K areas and inside the priority forest habitats located outside N2K sites, although forestry operations, such as logging, ditching, and suppression of natural regeneration, constitute a major threat to biodiversity (Bruun & Heilmann‐Clausen, 2021). The protected areas system in Sweden has recently been criticized for not meeting agreed‐on national and international forest biodiversity targets (Angelstam et al., 2020) and for not providing habitat for some forest bird species (Orlikowska et al., 2020). This might be because N2K sites are mostly concentrated in large contiguous areas in the northern boreal forest subregion, which hosts highly endangered habitats where productivity and diversity are low. Yet, only small and isolated patches are protected in species‐rich and productive regions from central and southern Sweden (Elbakidze et al., 2013). Similar patterns are observed on Macaronesian islands, a very diverse region that hosts a large number of endemic species in a limited space (Chiarucciet al., 2011; Whittaker et al., 2017) and is threatened by habitat fragmentation and loss, introduction of non‐native species, and climate change (Borges et al., 2020). Indeed, even if a large share of Macaronesia is already under protection (e.g., 46% of the Canary Islands is part of the N2K network), most protected land in this biogeographical region is limited to high elevations, which means that important vegetation types at low and middle elevations, including laurisilva, thermo‐sclerophyllous woodland, and *Euphorbia* scrub communities, are underprotected and receding (del Arco Aguilar et al., 2010).

Under an area‐based conservation framework, the amount of protected area is a fundamental target (Hoffmann, 2022; Maxwell et al., 2020) and therefore needs to be achieved. Yet, ensuring the representativeness of species and habitats is key to effective conservation initiatives. Logical evidence rooted on the species–area relationship calls for a carefully planned strategy to expand the network of protected areas, which should happen within a systematic conservation planning framework (e.g., Margules & Pressey, 2000). Indeed, although the amount of area is a fundamental determinant of the effectiveness of a protected area network, as predicted by classical biogeographical and macroecological theory (Lomolino, 2001; Santini et al., 2021; Scheiner et al., 2011), complex interactions between area and environmental heterogeneity are expected (Stein et al., 2014). In some cases, environmental heterogeneity can be even more important as a predictor of species richness than area per se (Malanson et al., 2023; Udy et al., 2021). This can be particularly true in human‐dominated landscapes, such as those that prevail in most of Europe. This points to the need for prioritizing environmental heterogeneity—including geodiversity (Gray, 2013)—in area‐based conservation planning to maximize the number of species under protection. Discussing specific strategies for the further developments of the N2K network is beyond the scope of this work, but surely maximizing environmental heterogeneity represented within the N2K network is key to increasing the proportion of species protected. This issue should be implicitly considered for achieving the objective of expanding protected areas in the EU to 30% of the land area, as mandated by the EU's Biodiversity Strategy for 2030 and the Kunming–Montreal global biodiversity framework (CBD, 2022).

The EVA database represents the best available source of plant co‐occurrence information at the European extent (Chytrý et al., 2016). However, it does not cover all plant diversity on the continent and consists of a mixture of different datasets, mostly collected with a preferential sampling approach. Many priority species are missing from the EVA dataset. In our selected sample of plots, we found only 38% of the plant species listed in the Habitats Directive, meaning that a substantial proportion (62%) of these legally protected species remain unrecorded in the EVA. This suggests the existence of not only a conservation gap, but also a knowledge gap regarding plant communities associated with these species because it is less likely for rare and uncommon species to be recorded in vegetation plots. Addressing this gap should be a monitoring priority. Targeted sampling efforts are needed to ensure that the remaining 62% of Habitat Directive species are adequately represented in the EVA plot network. These limitations are inherent to most biodiversity databases for large geographical scales that are assembled from different datasets. Even though some of the individual datasets are based on probabilistic samples, their assemblage cannot be considered unbiased, which creates theoretical challenges when trying to generalize observed patterns to the entire sampling population (e.g., Alessi et al., 2023; Chiarucci et al., 2011). Notwithstanding these limitations, recent macroecological studies show that these data can be employed successfully to describe plant diversity patterns at broad spatial extents as long as adequate resampling and cross‐validation techniques are used (e.g., Cao Pinna et al., 2024; Sabatini et al., 2022; Sporbert et al., 2019; Testolin et al., 2021; Večeřa et al., 2019; Xu et al., 2012).

The density of plots recorded per country inside and outside the N2K network was quite balanced in our study, except for a few combinations of countries and biogeographical regions for which the density of plots inside N2K sites was much lower than outside (Macaronesian region of Portugal, Mediterranean region of Croatia, Black Sea region of Romania, and Alpine region of Finland) and vice versa (Continental and Mediterranean regions of Italy, Atlantic region of France, Alpine regions of Bulgaria and Croatia). Therefore, results pertaining to such spatial units should be interpreted with caution. Although the general balance in plot density ensured a fair comparison of the plant diversity inside and outside the N2K network, designing and implementing standardized data collection across member states, together with data collection initiatives aimed at filling the many data gaps, are clear priorities (Moersberger et al., 2024; Pereira et al., 2022). Additional sampling effort for monitoring priority species and their habitats is needed to overcome the intrinsic limitations of preferential data collection and to better inform conservation planning and measures in the future. Moreover, expanding the analyses considering different levels of protection and including other types of protected areas can enhance knowledge of the roles of different area‐based conservation strategies in preserving biodiversity.

Our work here represents a necessary initial step in assessing the effectiveness of the N2K network in protecting plant diversity. Although over 85% of native species were also found outside the network, the latter encompassed nearly 90% of plant species—a remarkable result given that the network currently covers <20% of the EU land area. Yet, variations across countries and biogeographical regions were significant, indicating that the N2K network is not equally representative of different fractions of the gamma diversity of vascular plants across the EU. These results point to different interpretation and implementation strategies of the Habitats and Birds Directives across member states in terms of the proportion of land under protection and its management. In the context of the planned expansion of the N2K network to 30% of land area—as required by the EU 2030 Biodiversity Strategy—our results highlight the importance of data‐driven procedures to pinpoint underrepresented species and habitat types that should be included in the N2K network.

## Supporting information



Appendix S1. Surface area, number and density of vegetation plots from the European Vegetation Archive found inside and outside the Natura 2000 network for each combination of country and biogeographical region.
**Appendix S2**. Aggregated representation of sampling effort over Europe. Colors correspond to plot counts inside each hexagonal bin.
**Appendix S3**. Pairwise scatterplots and Pearson's correlation coefficients between overall species richness directly observed using vegetation plots from the European Vegetation Archive (Eva), estimated with the Michaelis‐Menten (Micmen), Asymptotic (Asymp) or Chao2 estimators, and derived using independent GBIF observations (Gbif) and the environmentally matched subset (Matched)
**Appendix S4**. Species listed in the last EEA report (EEA, 2020) but not found in the EVA database, reported in alphabetical order.
**Appendix S5**. Conservation priority species as defined by the EU Habitats Directive detected in some combinations of country and biogeographical region using vegetation plots from the European Vegetation Archive but missing from the EEA report (EEA, 2021).
**Appendix S6**. Percentage (%) of priority species found exclusively within, exclusively outside, and shared between areas inside and outside the Natura 2000 network compared to the total reported by the EEA (also including unreported priority species that were only found in the EVA dataset) for each combination of country and biogeographical region of the EU (A).
**Appendix S7**. Percentage (%) of native and priority species found exclusively within, exclusively outside, and shared between areas inside and outside the Natura 2000 network for each combination of country and biogeographical region of the EU.
**Appendix S8**. Percentage (%) of native species found within of the Natura 2000 network versus the percentage of land surface covered by the N2K network for each EU country, biogeographical region and their combination of country and biogeographical region of the EU.
**Appendix S9**. Percentage (%) of priority species found exclusively within, exclusively outside, and shared between areas inside and outside the Natura 2000 network compared to the total reported by the EEA for each combination of country and biogeographical region of the EU. Abbreviations: TOT—Total. See Figure 1 for additional abbreviations.
