# Supplementary material for: Representativeness of the Natura 2000 network for preserving plant biodiversity in the European Union
Source: Conserv Biol. 2025 Oct 12;40(2):e70158. doi: 10.1111/cobi.70158 (PMC13036301; doi:10.1111/cobi.70158)
Supplement: Supplementary file 1 — Appendix S1. Surface area, number and density of vegetation plots from the European Vegetation Archive found inside and outside the Natura 2000 network for each combination of country and biogeographical region. Appendix S2. Aggregated representation of sampling effort over Europe. Colors correspond to plot counts inside each hexagonal bin. Appendix S3. Pairwise scatterplots and Pearson's correlation coefficients between overall species richness directly observed using vegetation plots from the European Vegetation Archive (Eva), estimated with the Michaelis‐Menten (Micmen), Asymptotic (Asymp) or Chao2 estimators, and derived using independent GBIF observations (Gbif) and the environmentally matched subset (Matched) Appendix S4. Species listed in the last EEA report (EEA, 2020) but not found in the EVA database, reported in alphabetical order. Appendix S5. Conservation priority species as defined by the EU Habitats Directive detected in some combinations of country and biogeographical region using vegetation plots from the European Vegetation Archive but missing from the EEA report (EEA, 2021). Appendix S6. Percentage (%) of priority species found exclusively within, exclusively outside, and shared between areas inside and outside the Natura 2000 network compared to the total reported by the EEA (also including unreported priority species that were only found in the EVA dataset) for each combination of country and biogeographical region of the EU (A). Appendix S7. Percentage (%) of native and priority species found exclusively within, exclusively outside, and shared between areas inside and outside the Natura 2000 network for each combination of country and biogeographical region of the EU. Appendix S8. Percentage (%) of native species found within of the Natura 2000 network versus the percentage of land surface covered by the N2K network for each EU country, biogeographical region and their combination of country and biogeographical region of the EU. Appendix S9. Pe [file COBI-40-e70158-s001.docx]

**Supporting information for “Representativeness of the Natura 2000 network for preserving plant biodiversity in the European Union”**

**Appendix S1.** Surface area, number and density of vegetation plots from the European Vegetation Archive found inside and outside the Natura 2000 network for each combination of country and biogeographical region.

| **Countries** | **Biogeographical regions** | **Area** | | **Number of plots** | | **Plot density**  **(n/km^2^)** | |
| --- | --- | --- | --- | --- | --- | --- | --- |
|  |  | ***Inside*** | ***Outside*** | ***Inside*** | ***Outside*** | ***Inside*** | ***Outside*** |
| Austria | Alpine | 8676.88 | 43996.72 | 2007 | 5326 | 0.23 | 0.12 |
| Austria | Continental | 4302.60 | 26813.26 | 1539 | 2850 | 0.36 | 0.11 |
| Belgium | Atlantic | 1772.94 | 17001.33 | 5587 | 2990 | 3.15 | 0.18 |
| Belgium | Continental | 2111.66 | 9709.43 | 73 | 130 | 0.03 | 0.01 |
| Bulgaria | Black Sea | 3985.32 | 3287.27 | 322 | 76 | 0.08 | 0.02 |
| Bulgaria | Alpine | 10891.74 | 6432.18 | 4233 | 438 | 0.39 | 0.07 |
| Bulgaria | Continental | 23819.79 | 63469.50 | 1556 | 2885 | 0.07 | 0.05 |
| Croatia | Mediterranean | 7117.27 | 8318.40 | 231 | 1076 | 0.03 | 0.13 |
| Croatia | Continental | 7262.07 | 23124.68 | 649 | 2309 | 0.09 | 0.1 |
| Croatia | Alpine | 4964.19 | 3348.63 | 812 | 14 | 0.16 | 0 |
| Cyprus | Mediterranean | 1615.26 | 3727.04 | 16 | 17 | 0.01 | 0 |
| Czech Republic | Continental | 10577.63 | 64728.93 | 13418 | 48229 | 1.27 | 0.75 |
| Czech Republic | Pannonian | 515.55 | 2873.38 | 493 | 2314 | 0.96 | 0.81 |
| Denmark | Atlantic | 1148.94 | 11974.62 | 40388 | 26873 | 35.15 | 2.24 |
| Denmark | Continental | 1886.55 | 26523.53 | 102203 | 63796 | 54.17 | 2.41 |
| Estonia | Boreal | 7754.33 | 37098.06 | 256 | 988 | 0.03 | 0.03 |
| Finland | Boreal | 29742.13 | 285719.31 | 73 | 1967 | 0 | 0.01 |
| Finland | Alpine | 11758.35 | 3837.31 | 319 | 1128 | 0.03 | 0.29 |
| France | Continental | 21617.58 | 161903.54 | 3527 | 3665 | 0.16 | 0.02 |
| France | Mediterranean | 15624.28 | 49561.50 | 2859 | 2603 | 0.18 | 0.05 |
| France | Atlantic | 21563.44 | 245198.24 | 4374 | 2936 | 0.2 | 0.01 |
| France | Alpine | 10467.85 | 20229.47 | 4928 | 2598 | 0.47 | 0.13 |
| Germany | Continental | 46793.83 | 235899.93 | 9138 | 16782 | 0.2 | 0.07 |
| Germany | Atlantic | 6169.65 | 63801.64 | 2328 | 13645 | 0.38 | 0.21 |
| Germany | Alpine | 1644.06 | 2635.90 | 435 | 288 | 0.26 | 0.11 |
| Greece | Mediterranean | 34611.35 | 94246.65 | 2996 | 2419 | 0.09 | 0.03 |
| Hungary | Pannonian | 19819.38 | 72910.76 | 287 | 179 | 0.01 | 0 |
| Ireland | Atlantic | 8790.92 | 59926.44 | 8876 | 7696 | 1.01 | 0.13 |
| Italy | Mediterranean | 31773.66 | 129471.28 | 14413 | 8412 | 0.45 | 0.06 |
| Italy | Continental | 8688.28 | 78518.57 | 3616 | 3546 | 0.42 | 0.05 |
| Italy | Alpine | 16216.09 | 34595.57 | 4228 | 3474 | 0.26 | 0.10 |
| Latvia | Boreal | 7316.37 | 56965.26 | 3659 | 3175 | 0.5 | 0.06 |
| Lithuania | Boreal | 7897.63 | 56258.07 | 0 | 442 | 0 | 0.01 |
| Luxembourg | Continental | 642.17 | 1966.30 | 5 | 7 | 0.01 | 0 |
| Netherlands | Atlantic | 5236.70 | 31554.77 | 44179 | 85845 | 8.44 | 2.72 |
| Poland | Continental | 55893.40 | 245925.52 | 8134 | 22248 | 0.15 | 0.09 |
| Poland | Alpine | 5152.90 | 5209.99 | 2116 | 774 | 0.41 | 0.15 |
| Portugal | Macaronesian | 516.80 | 2303.58 | 4 | 195 | 0.01 | 0.08 |
| Portugal | Mediterranean | 16665.95 | 66091.92 | 505 | 777 | 0.03 | 0.01 |
| Portugal | Atlantic | 883.81 | 3844.35 | 51 | 14 | 0.06 | 0 |
| Romania | Steppic | 7726.64 | 29011.56 | 209 | 393 | 0.03 | 0.01 |
| Romania | Black Sea | 2103.17 | 1488.17 | 72 | 205 | 0.03 | 0.14 |
| Romania | Pannonian | 2261.40 | 13099.44 | 168 | 343 | 0.07 | 0.03 |
| Romania | Continental | 21070.17 | 109813.35 | 667 | 4209 | 0.03 | 0.04 |
| Romania | Alpine | 19087.17 | 29962.22 | 3265 | 1525 | 0.17 | 0.05 |
| Slovak Republic | Alpine | 11529.71 | 22590.89 | 8903 | 5919 | 0.77 | 0.26 |
| Slovak Republic | Pannonian | 2722.41 | 11532.44 | 449 | 2316 | 0.16 | 0.20 |
| Slovenia | Continental | 3467.57 | 9067.58 | 593 | 2217 | 0.17 | 0.24 |
| Slovenia | Alpine | 4207.46 | 3544.46 | 3141 | 1270 | 0.75 | 0.36 |
| Spain | Alpine | 4942.94 | 4275.67 | 2199 | 761 | 0.44 | 0.18 |
| Spain | Mediterranean | 116194.82 | 317446.31 | 11291 | 9806 | 0.1 | 0.03 |
| Spain | Atlantic | 13592.77 | 41695.69 | 4865 | 6156 | 0.36 | 0.15 |
| Spain | Macaronesian | 3221.06 | 3667.04 | 869 | 266 | 0.27 | 0.07 |
| Sweden | Boreal | 15359.16 | 328147.15 | 56 | 2720 | 0 | 0.01 |
| Sweden | Continental | 836.39 | 14496.46 | 5 | 349 | 0.01 | 0.02 |
| Sweden | Alpine | 38152.62 | 46918.63 | 119 | 127 | 0 | 0 |


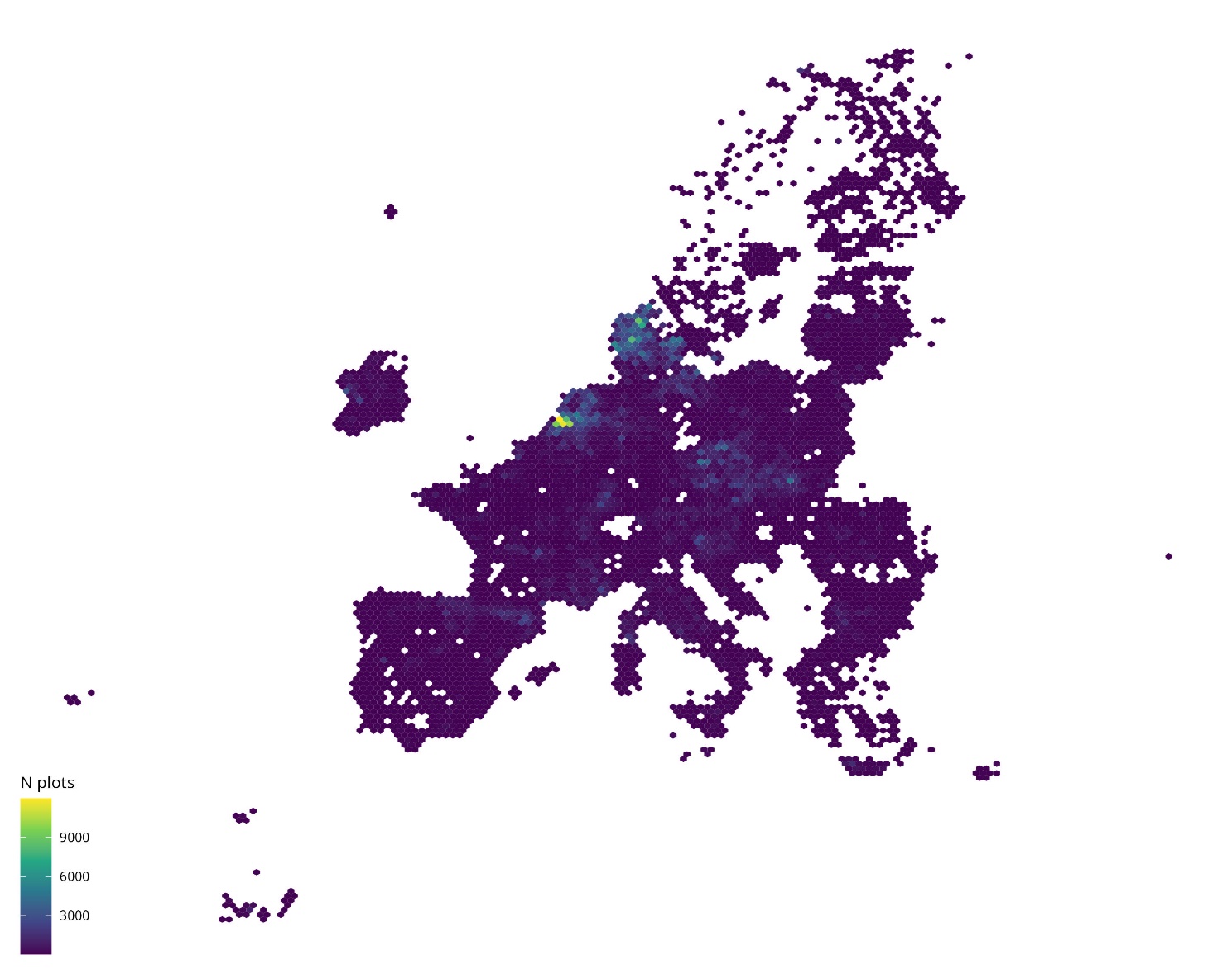


**Appendix S2.** Aggregated representation of sampling effort over Europe. Colours correspond to plot counts inside each hexagonal bin.


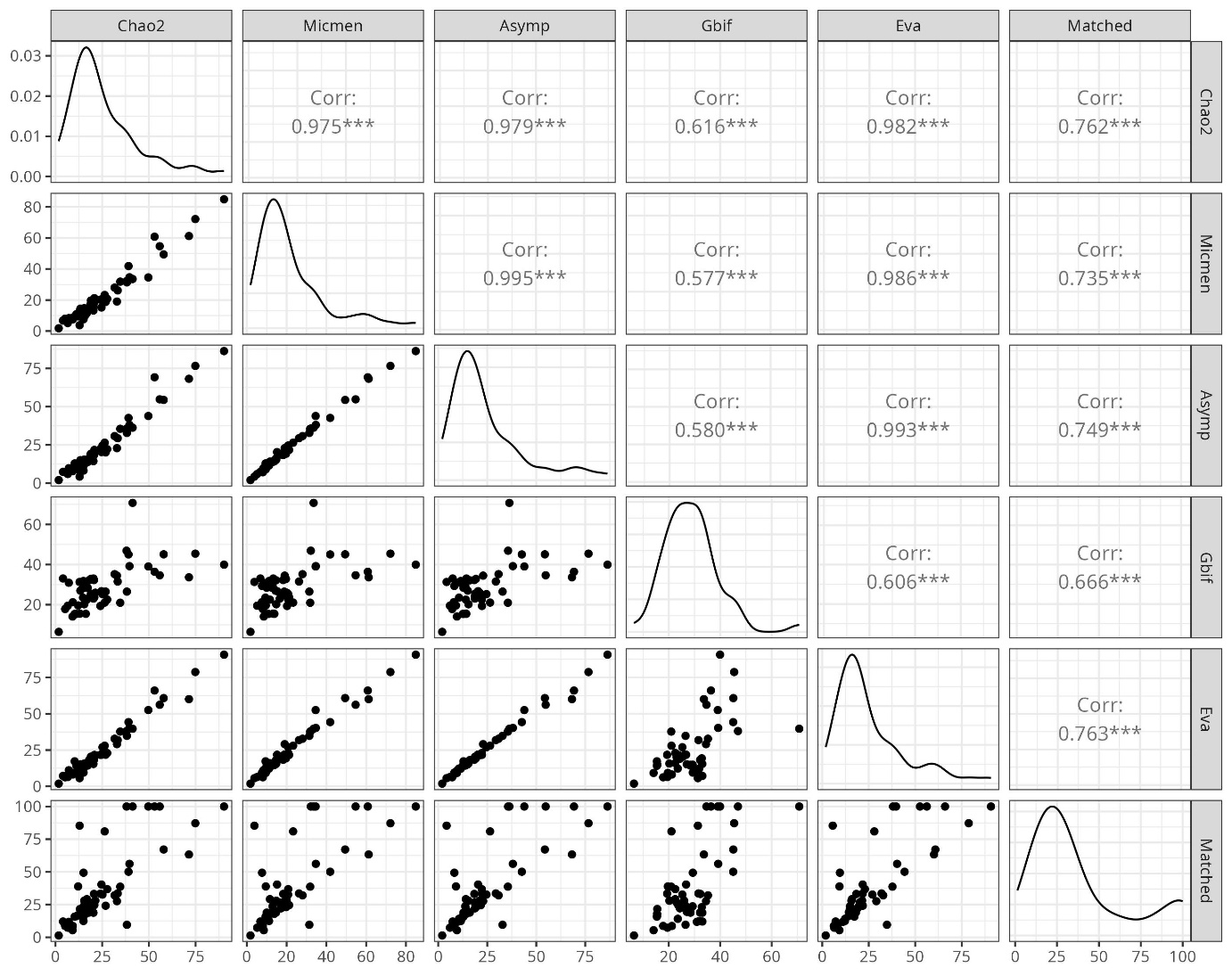


**Appendix S3.** Pairwise scatterplots and Pearson’s correlation coefficients between overall species richness directly observed using vegetation plots from the European Vegetation Archive (Eva), estimated with the Michaelis-Menten (Micmen), Asymptotic (Asymp) or Chao2 estimators, and derived using independent GBIF observations (Gbif) and the environmentally matched subset (Matched). Significance code: *** p < 0.001

**Appendix S4.** Species listed in the last EEA report (EEA, 2020) but not found in the EVA database, reported in alphabetical order.

| **Missing Habitats Directive plant species** | | | |
| --- | --- | --- | --- |
| ***Ac-Ce*** | ***Ch-Ib*** | ***Ib-Pl*** | ***Pl-Vi*** |
| Acis nicaeensis | Chaenorhinum serpyllifolium | Iberis runemarkii | Plantago malato-belizii |
| Aconitum corsicum | Chaerophyllum azoricum | Ionopsidium savianum | Platanthera obtusata |
| Aconitum firmum | Cheirolophus duranii | Iris lusitanica | Poa granitica |
| Aeonium saundersii | Cheirolophus junonianus | Iris marsica | Polygonum praelongum |
| Aichryson dumosum | Cheirolophus massonianus | Isoetes boryana | Polystichum drepanum |
| Alisma wahlenbergii | Chionodoxa lochiae | Isoetes malinverniana | Potentilla emilii-popii |
| Allium grosii | Cirsium latifolium | Isoplexis chalcantha | Primula apennina |
| Ammi trifoliatum | Cistus palhinhae | Isoplexis isabelliana | Primula scandinavica |
| Anarrhinum longipedicellatum | Clinopodium taygeteum | Jacobaea vulgaris | Prunus azorica |
| Androcymbium europaeum | Cochlearia polonica | Jasminum azoricum | Puccinellia phryganodes |
| Androcymbium psammophilum | Coincya rupestris | Juncus valvatus | Puccinellia pungens |
| Androcymbium rechingeri | Colchicum arenarium | Jurinea fontqueri | Pulsatilla grandis |
| Angelica lignescens | Consolida samia | Kunkeliella subsucculenta | Pulsatilla slavica |
| Anthyllis hystrix | Convolvulus fernandesii | Lactuca watsoniana | Pulsatilla subslavica |
| Antirrhinum charidemi | Convolvulus massonii | Laserpitium longiradium | Pulsatilla vulgaris |
| Antirrhinum lopesianum | Coronopus navasii | Leopoldia gussonei | Pyrus magyarica |
| Apium bermejoi | Corydalis gotlandica | Leuzea longifolia | Ranunculus kykkoensis |
| Aquilegia bertolonii | Crambe arborea | Limonium arborescens | Ranunculus weyleri |
| Aquilegia pyrenaica | Crambe laevigata | Limonium dendroides | Reseda decursiva |
| Arabis kennedyae | Crambe sventenii | Limonium dodartii | Rhaponticoides fraylensis |
| Arabis sadina | Cremnophyton lanfrancoi | Limonium insulare | Rhinanthus osiliensis |
| Arceuthobium azoricum | Crepis granatensis | Limonium lanceolatum | Rhynchosinapis erucastrum |
| Arctagrostis latifolia | Crepis tectorum | Limonium multiflorum | Ribes sardoum |
| Arctophila fulva | Crocus cyprius | Limonium pseudolaetum | Rosmarinus tomentosus |
| Arenaria ciliata | Crocus hartmannianus | Limonium spectabile | Rumex azoricus |
| Arenaria humifusa | Cyclamen fatrense | Limonium sventenii | Salvia veneris |
| Arenaria nevadensis | Dactylorhiza kalopissii | Linaria algarviana | Sambucus palmensis |
| Argyranthemum lidii | Daphne petraea | Linaria ficalhoana | Santolina impressa |
| Argyranthemum pinnatifidum | Daphne rodriguezii | Linaria hellenica | Saussurea alpina |
| Argyranthemum thalassophilum | Delphinium caseyi | Linaria ricardoi | Saxifraga cintrana |
| Argyranthemum winteri | Dendriopoterium pulidoi | Linaria tonzigii | Saxifraga portosanctana |
| Armeria berlengensis | Dianthus arenarius | Linum dolomiticum | Saxifraga presolanensis |
| Armeria helodes | Dianthus cintranus | Lotus azoricus | Saxifraga tombeanensis |
| Armeria neglecta | Dianthus laricifolius | Lotus callis-viridis | Saxifraga vayredana |
| Armeria sampaioi | Dianthus lumnitzeri | Lotus kunkelii | Scabiosa nitens |
| Artemisia campestris | Dianthus plumarius | Luzula arctica | Scilla beirana |
| Artemisia eriantha | Diplazium sibiricum | Lythrum flexuosum | Scilla morrisii |
| Artemisia laciniata | Diplotaxis ibicensis | Marcetella maderensis | Scrophularia grandiflora |
| Artemisia oelandica | Diplotaxis siettiana | Marsilea azorica | Semele maderensis |
| Asphodelus bento-rainhae | Diplotaxis siifolia | Marsilea batardae | Senecio caespitosus |
| Astragalus algarbiensis | Doronicum plantagineum | Melanoselinum decipiens | Senecio elodes |
| Astragalus alopecurus | Draba cacuminum | Minuartia smejkalii | Senecio lagascanus |
| Astragalus maritimus | Draba cinerea | Moehringia jankae | Sideritis cypria |
| Astragalus peterfii | Draba dorneri | Moehringia lateriflora | Sideritis cystosiphon |
| Asyneuma giganteum | Echium candicans | Moehringia tommasinii | Sideritis discolor |
| Athamanta cortiana | Erica azorica | Monanthes wildpretii | Sideritis incana |
| Atractylis arbuscula | Erodium astragaloides | Monizia edulis | Sideritis infernalis |
| Atractylis preauxiana | Erodium paularense | Musschia wollastonii | Sideritis javalambrensis |
| Avenula hackelii | Erodium rupicola | Myosotis azorica | Sideritis marmorea |
| Azorina vidalii | Erucastrum palustre | Myosotis lusitanica | Sideritis serrata |
| Bellevalia hackelii | Eryngium viviparum | Myosotis maritima | Silene hicesiae |
| Bencomia brachystachya | Erysimum pieninicum | Myrica rivas-martinezii | Silene hifacensis |
| Bencomia sphaerocarpa | Euphorbia bourgeana | Najas tenuissima | Silene holzmannii |
| Berberis maderensis | Euphorbia handiensis | Narcissus cavanillesii | Silene mariana |
| Beta patula | Euphorbia margalidiana | Narcissus fernandesii | Silene orphanidis |
| Biscutella neustriaca | Euphorbia stygiana | Narcissus pseudonarcissus | Silene rothmaleri |
| Biscutella sempervirens | Euphorbia transtagana | Narcissus scaberulus | Sinapidendron rupestre |
| Borderea chouardii | Euphrasia azorica | Narcissus segurensis | Sisymbrium cavanillesianum |
| Brassica glabrescens | Euphrasia grandiflora | Narcissus viridiflorus | Sisymbrium supinum |
| Brassica hilarionis | Euphrasia marchesettii | Narcissus yepesii | Solanum lidii |
| Braya linearis | Euphrasia nana | Naufraga balearica | Solenanthus albanicus |
| Bromus grossus | Ferula latipinna | Nepeta argolica | Sorbus maderensis |
| Bunium brevifolium | Fritillaria conica | Omphalodes kuzinskyanae | Sorbus teodori |
| Bupleurum capillare | Fritillaria gussichiae | Ononis maweana | Stemmacantha cynaroides |
| Calamagrostis chalybaea | Fritillaria obliqua | Onopordum carduelium | Stipa bavarica |
| Calendula maderensis | Fritillaria rhodocanakis | Onopordum nogalesii | Stipa danubialis |
| Calypso bulbosa | Galium cracoviense | Onosma tornensis | Stipa veneta |
| Caralluma burchardii | Galium litorale | Ophioglossum polyphyllum | Sventenia bupleuroides |
| Carduus myriacanthus | Gaudinia hispanica | Ophrys argolica | Symphytum cycladense |
| Carex malato-belizii | Gentianella anglica | Ophrys kotschyi | Syringa josikaea |
| Carex panormitana | Gentianella bohemica | Orchis scopulorum | Tanacetum ptarmiciflorum |
| Carlina onopordifolia | Geranium maderense | Ornithogalum reverchonii | Tephroseris longifolia |
| Centaurea akamantis | Globularia ascanii | Orobanche densiflora | Teucrium abutiloides |
| Centaurea attica | Globularia sarcophylla | Paeonia cambessedesii | Teucrium charidemi |
| Centaurea balearica | Goodyera macrophylla | Paeonia clusii | Teucrium lepicephalum |
| Centaurea borjae | Gypsophila papillosa | Paeonia officinalis | Teucrium turredanum |
| Centaurea heldreichii | Halimium verticillatum | Paeonia parnassica | Thymelaea broteriana |
| Centaurea horrida | Helianthemum alypoides | Palaeocyanus crassifolius | Thymus camphoratus |
| Centaurea jankae | Helianthemum bystropogophyllum | Papaver radicatum | Thymus carnosus |
| Centaurea kalambakensis | Helichrysum melitense | Parolinia schizogynoides | Thymus villosus |
| Centaurea kartschiana | Helichrysum sibthorpii | Pericallis hadrosoma | Tozzia carpathica |
| Centaurea lactiflora | Herniaria algarvica | Petagnaea gussonei | Tripolium sorrentinoi |
| Centaurea micrantha | Herniaria litardierei | Petrocoptis grandiflora | Trisetum subalpestre |
| Centaurea niederi | Herniaria lusitanica | Petrocoptis montsicciana | Tulipa cypria |
| Centaurea peucedanifolia | Himantoglossum jankae | Petrocoptis pseudoviscosa | Ulex densus |
| Centaurea pinnata | Hippuris tetraphylla | Phalaris maderensis | Veronica oetaea |
| Centaurea pontica | Holcus setiglumis | Phlomis brevibracteata | Vicia bifoliolata |
| Centaurea princeps | Hormathophylla pyrenaica | Phlomis cypria | Vincetoxicum pannonicum |
| Centaurea pulvinata | Hyacinthoides vincentina | Picris willkommii | Viola athois |
| Centaurium rigualii | Hymenostemma pseudanthemis | Pinguicula crystallina | Viola cazorlensis |
| Centaurium somedanum | Hyoseris frutescens | Pittosporum coriaceum | Viola delphinantha |
| Centranthus amazonum | Hypericum aciferum | Plantago algarbiensis | Viola jaubertiana |
| Ceropegia dichotoma | Iberis procumbens | Plantago almogravensis | Viola paradoxa |

**Appendix S5.** Conservation priority species as defined by the EU Habitats Directive detected in some combinations of country and biogeographical region using vegetation plots from the European Vegetation Archive but missing from the EEA report (EEA, 2021).

| **Country** | **Biogeographical region** | **Species** |
| --- | --- | --- |
| Bulgaria | BLS | Klasea lycopifolia |
| Bulgaria | CON | Stipa zalesskii |
| Bulgaria | CON | Tulipa hungarica |
| Croatia | MED | Aquilegia kitaibelii |
| Croatia | MED | Asplenium adulterinum |
| Croatia | MED | Cerastium dinaricum |
| Czech Republic | CON | Lycopodium zeilleri |
| Denmark | ATL | Carex holostoma |
| France | ATL | Anchusa crispa |
| France | ATL | Centaurea corymbosa |
| France | ATL | Herniaria maritima |
| France | CON | Centaurea corymbosa |
| Germany | ATL | Myosotis rehsteineri |
| Germany | CON | Lycopodium zeilleri |
| Greece | MED | Mandragora officinarum |
| Italy | ALP | Centranthus trinervis |
| Italy | ALP | Crocus etruscus |
| Italy | CON | Primula glaucescens |
| Italy | MED | Colchicum corsicum |
| Italy | MED | Linaria coutinhoi |
| Italy | MED | Spergularia azorica |
| Lithuania | CON | Linaria loeselii |
| Poland | ALP | Galium sudeticum |
| Portugal | MED | Oenanthe divaricata |
| Portugal | MED | Sibthorpia peregrina |
| Romania | CON | Arnica montana |
| Romania | CON | Campanula romanica |
| Romania | STE | Dianthus diutinus |
| Slovak Republic | CON | Cirsium brachycephalum |
| Slovak Republic | PAN | Dianthus nitidus |
| Slovenia | PAN | Eleocharis carniolica |
| Spain | ALP | Erigeron frigidus |
| Spain | ATL | Euphorbia nevadensis |
| Spain | ATL | Pseudarrhenatherum pallens |
| Spain | MAC | Oenanthe divaricata |
| Spain | MED | Festuca henriquesii |
| Spain | MED | Woodwardia radicans |

**
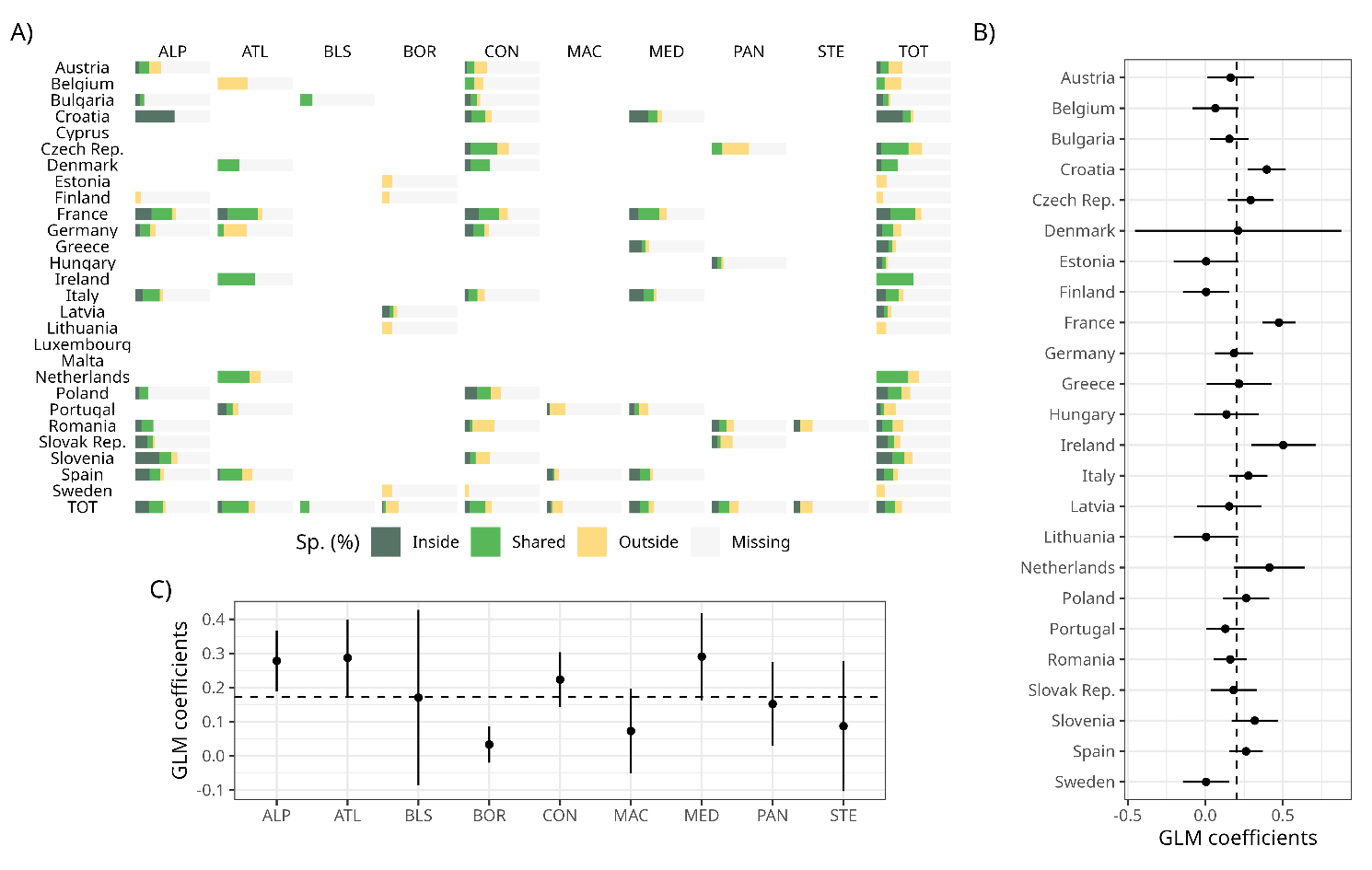
**

**Appendix S6.** Percentage (%) of priority species found exclusively within, exclusively outside, and shared between areas inside and outside the Natura 2000 network compared to the total reported by the EEA (also including unreported priority species that were only found in the EVA dataset) for each combination of country and biogeographical region of the EU (A). The % of missing species is also reported. GLM coefficients of the proportion of priority species found within the network against countries (B) and biogeographical regions (C) accounting for sampling effort. Black bars and dots indicate, respectively, the 95% confidence interval and the mean of the GLM coefficients. The dashed lines indicate the average pattern over Europe. Abbreviations: TOT - Total. See Figure 1 for additional abbreviations.

**Appendix S7.** Percentage (%) of native and priority species found exclusively within, exclusively outside, and shared between areas inside and outside the Natura 2000 network for each combination of country and biogeographical region of the EU. The percentage of land inside the network is also reported. Native species numbers were estimated using Chao2, based on the data contained in the European Vegetation Archive. Priority species numbers are based on raw counts. NAs indicate missing values in a particular combination of country and biogeographical region.

|  | **Native species** | | | **Priority species** | | | | **% land in N2K** |
| --- | --- | --- | --- | --- | --- | --- | --- | --- |
|  | ***Shared*** | ***Inside*** | ***Outside*** | ***Shared*** | ***Inside*** | ***Outside*** | ***Missing*** |  |
| **Austria** | | | | | | | |  |
| TOT | 73.84 | 7.75 | 18.41 | 13.51 | 8.11 | 18.92 | 59.46 | 0.15 |
| ALP | 69.69 | 11.35 | 18.96 | 15.38 | 11.54 | 15.38 | 57.69 | 0.16 |
| CON | 71.72 | 7.93 | 20.35 | 8.33 | 8.33 | 20.83 | 62.50 | 0.14 |
| **Belgium** | | | | | | | |  |
| TOT | 69.90 | 9.18 | 20.92 | 16.67 | 0.00 | 33.33 | 50.00 | 0.13 |
| ATL | 67.80 | 13.24 | 18.96 | 0.00 | 0.00 | 66.67 | 33.33 | 0.09 |
| CON | 66.63 | 0.46 | 32.91 | 20.00 | 0.00 | 20.00 | 60.00 | 0.18 |
| **Bulgaria** | | | | | | | |  |
| TOT | 71.03 | 20.02 | 8.96 | 8.00 | 12.00 | 4.00 | 76.00 | 0.35 |
| ALP | 54.48 | 38.94 | 6.58 | 8.33 | 8.33 | 0.00 | 83.33 | 0.63 |
| BLS | 38.98 | 55.84 | 5.19 | 20.00 | 0.00 | 0.00 | 80.00 | 0.55 |
| CON | 72.88 | 11.28 | 15.84 | 10.00 | 10.00 | 5.00 | 75.00 | 0.27 |
| **Croatia** | | | | | | | |  |
| TOT | 54.47 | 15.49 | 30.04 | 12.50 | 29.17 | 0.00 | 58.33 | 0.36 |
| ALP | 25.24 | 73.00 | 1.76 | 0.00 | 47.06 | 0.00 | 52.94 | 0.60 |
| CON | 59.20 | 9.13 | 31.66 | 25.00 | 12.50 | 0.00 | 62.50 | 0.24 |
| MED | 42.64 | 22.70 | 34.66 | 13.33 | 26.67 | 0.00 | 60.00 | 0.46 |
| **Cyprus** | | | | | | | |  |
| TOT | 52.12 | 12.66 | 35.22 | NA | NA | NA | NA | 0.30 |
| MED | 52.12 | 12.66 | 35.22 | NA | NA | NA | NA | 0.30 |
| **Czech** **Republic** | | | | | | | |  |
| TOT | 70.36 | 5.62 | 24.02 | 43.24 | 8.11 | 21.62 | 27.03 | 0.14 |
| CON | 67.82 | 5.32 | 26.87 | 43.75 | 9.38 | 18.75 | 28.12 | 0.14 |
| PAN | 61.38 | 5.31 | 33.31 | 15.38 | 0.00 | 38.46 | 46.15 | 0.15 |
| **Denmark** | | | | | | | |  |
| TOT | 77.73 | 6.64 | 15.63 | 37.50 | 12.50 | 0.00 | 50.00 | 0.07 |
| ATL | 74.18 | 10.80 | 15.02 | 50.00 | 0.00 | 0.00 | 50.00 | 0.09 |
| CON | 82.38 | 4.70 | 12.92 | 50.00 | 16.67 | 0.00 | 33.33 | 0.07 |
| **Estonia** | | | | | | | |  |
| TOT | 72.52 | 2.33 | 25.16 | 0.00 | 0.00 | 20.00 | 80.00 | 0.17 |
| BOR | 72.52 | 2.33 | 25.16 | 0.00 | 0.00 | 20.00 | 80.00 | 0.17 |
| **Finland** | | | | | | | |  |
| TOT | 54.74 | 1.86 | 43.40 | 0.00 | 0.00 | 13.33 | 86.67 | 0.13 |
| ALP | 84.66 | 5.06 | 10.28 | 0.00 | 0.00 | 12.50 | 87.50 | 0.75 |
| BOR | 39.29 | 2.78 | 57.92 | 0.00 | 0.00 | 14.81 | 85.19 | 0.09 |
| **France** | | | | | | | |  |
| TOT | 87.06 | 6.39 | 6.55 | 38.81 | 22.39 | 8.96 | 29.85 | 0.13 |
| ALP | 69.45 | 14.30 | 16.25 | 33.33 | 26.67 | 6.67 | 33.33 | 0.34 |
| ATL | 76.18 | 5.83 | 17.98 | 48.00 | 16.00 | 8.00 | 28.00 | 0.08 |
| CON | 83.14 | 9.71 | 7.15 | 36.84 | 26.32 | 15.79 | 21.05 | 0.12 |
| MED | 78.68 | 10.14 | 11.18 | 31.43 | 14.29 | 11.43 | 42.86 | 0.24 |
| **Germany** | | | | | | | |  |
| TOT | 70.72 | 9.17 | 20.10 | 17.86 | 10.71 | 17.86 | 53.57 | 0.15 |
| ALP | 66.76 | 18.18 | 15.06 | 28.57 | 14.29 | 14.29 | 42.86 | 0.38 |
| ATL | 60.22 | 0.68 | 39.10 | 11.11 | 0.00 | 44.44 | 44.44 | 0.09 |
| CON | 69.81 | 9.85 | 20.34 | 14.81 | 14.81 | 11.11 | 59.26 | 0.17 |
| **Greece** | | | | | | | |  |
| TOT | 51.97 | 27.21 | 20.81 | 5.00 | 16.67 | 5.00 | 73.33 | 0.27 |
| MED | 51.97 | 27.21 | 20.81 | 5.00 | 16.67 | 5.00 | 73.33 | 0.27 |
| **Hungary** | | | | | | | |  |
| TOT | 48.53 | 34.66 | 16.82 | 5.88 | 8.82 | 2.94 | 82.35 | 0.21 |
| PAN | 48.53 | 34.66 | 16.82 | 5.88 | 8.82 | 2.94 | 82.35 | 0.21 |
| **Ireland** | | | | | | | |  |
| TOT | 76.64 | 8.38 | 14.98 | 100.00 | 0.00 | 0.00 | 0.00 | 0.13 |
| ATL | 76.64 | 8.38 | 14.98 | 100.00 | 0.00 | 0.00 | 0.00 | 0.13 |
| **Italy** | | | | | | | |  |
| TOT | 78.56 | 11.39 | 10.04 | 16.83 | 12.87 | 4.95 | 65.35 | 0.19 |
| ALP | 69.76 | 15.49 | 14.75 | 24.39 | 12.20 | 2.44 | 60.98 | 0.32 |
| CON | 77.23 | 9.43 | 13.34 | 11.43 | 5.71 | 8.57 | 74.29 | 0.10 |
| MED | 72.95 | 17.75 | 9.29 | 14.06 | 18.75 | 4.69 | 62.50 | 0.20 |
| **Latvia** | | | | | | | |  |
| TOT | 73.03 | 22.92 | 4.05 | 6.67 | 13.33 | 6.67 | 73.33 | 0.11 |
| BOR | 73.03 | 22.92 | 4.05 | 6.67 | 13.33 | 6.67 | 73.33 | 0.11 |
| **Lithuania** | | | | | | | |  |
| TOT | 0.00 | 0.00 | 100.00 | 0.00 | 0.00 | 16.67 | 83.33 | 0.12 |
| BOR | 0.00 | 0.00 | 100.00 | 0.00 | 0.00 | 16.67 | 83.33 | 0.12 |
| **Luxembourg** | | | | | | | |  |
| TOT | 32.41 | 0.00 | 71.44 | NA | NA | NA | NA | 0.25 |
| CON | 32.41 | 0.00 | 71.44 | NA | NA | NA | NA | 0.25 |
| **Malta** | | | | | | | |  |
|  | NA | NA | NA | NA | NA | NA | NA | NA |
| **Netherlands** | | | | | | | |  |
| TOT | 76.82 | 5.92 | 17.25 | 75.00 | 0.00 | 25.00 | 0.00 | 0.14 |
| ATL | 76.82 | 5.92 | 17.25 | 75.00 | 0.00 | 25.00 | 0.00 | 0.14 |
| **Poland** | | | | | | | |  |
| TOT | 66.28 | 10.09 | 23.64 | 21.05 | 15.79 | 13.16 | 50.00 | 0.20 |
| ALP | 61.06 | 26.81 | 12.14 | 15.38 | 7.69 | 0.00 | 76.92 | 0.50 |
| CON | 66.57 | 5.73 | 27.70 | 22.58 | 16.13 | 16.13 | 45.16 | 0.19 |
| **Portugal** | | | | | | | |  |
| TOT | 54.45 | 13.19 | 32.36 | 1.78 | 4.14 | 1.78 | 92.31 | 0.20 |
| ATL | 50.76 | 31.77 | 17.47 | 9.09 | 4.55 | 4.55 | 81.82 | 0.19 |
| MAC | 5.26 | 4.11 | 90.63 | NA | NA | NA | NA | 0.18 |
| MED | 59.45 | 14.20 | 26.35 | 1.94 | 5.83 | 2.91 | 89.32 | 0.20 |
| **Romania** | | | | | | | |  |
| TOT | 65.14 | 13.62 | 21.23 | 15.91 | 9.09 | 15.91 | 59.09 | 0.22 |
| ALP | 57.86 | 23.40 | 18.74 | 21.05 | 10.53 | 0.00 | 68.42 | 0.39 |
| BLS | 30.99 | 13.21 | 55.80 | NA | NA | NA | NA | 0.59 |
| CON | 55.59 | 4.78 | 39.63 | 3.45 | 6.90 | 31.03 | 58.62 | 0.16 |
| PAN | 52.15 | 6.56 | 41.29 | 10.00 | 10.00 | 10.00 | 70.00 | 0.15 |
| STE | 29.73 | 9.04 | 61.24 | 0.00 | 8.33 | 16.67 | 75.00 | 0.21 |
| **Slovak** **Republic** | | | | | | | |  |
| TOT | 70.10 | 12.07 | 17.82 | 9.76 | 19.51 | 9.76 | 60.98 | 0.29 |
| ALP | 65.10 | 14.27 | 20.62 | 10.34 | 20.69 | 3.45 | 65.52 | 0.34 |
| PAN | 58.99 | 2.97 | 38.05 | 4.17 | 8.33 | 16.67 | 70.83 | 0.19 |
| **Slovenia** | | | | | | | |  |
| TOT | 62.48 | 19.02 | 18.49 | 15.62 | 25.00 | 12.50 | 46.88 | 0.38 |
| ALP | 60.92 | 29.93 | 9.16 | 15.00 | 40.00 | 10.00 | 35.00 | 0.54 |
| CON | 52.40 | 9.37 | 38.24 | 8.70 | 8.70 | 21.74 | 60.87 | 0.28 |
| **Spain** | | | | | | | |  |
| TOT | 73.07 | 11.29 | 15.64 | 9.28 | 8.25 | 5.15 | 77.32 | 0.27 |
| ALP | 67.43 | 17.16 | 15.41 | 17.65 | 23.53 | 5.88 | 52.94 | 0.54 |
| ATL | 61.94 | 16.71 | 21.35 | 24.24 | 6.06 | 12.12 | 57.58 | 0.25 |
| MAC | 53.22 | 14.43 | 32.35 | 0.00 | 6.56 | 8.20 | 85.25 | 0.47 |
| MED | 73.90 | 12.98 | 13.11 | 9.76 | 9.76 | 3.25 | 77.24 | 0.27 |
| **Sweden** | | | | | | | |  |
| TOT | 33.64 | 3.47 | 62.89 | 0.00 | 0.00 | 15.91 | 84.09 | 0.12 |
| ALP | 40.00 | 35.38 | 24.62 | NA | NA | NA | NA | 0.45 |
| BOR | 25.50 | 0.00 | 74.85 | 0.00 | 0.00 | 18.75 | 81.25 | 0.04 |
| CON | 8.15 | 1.63 | 90.21 | 0.00 | 0.00 | 9.09 | 90.91 | 0.05 |
| **Total** | | | | | | | |  |
| ALP | 75.20 | 15.40 | 9.40 | 19.49 | 20.34 | 4.24 | 55.93 | 0.39 |
| ATL | 73.60 | 10.02 | 16.38 | 36.84 | 10.53 | 8.77 | 43.86 | 0.11 |
| BLS | 48.27 | 44.57 | 7.16 | 14.29 | 0.00 | 0.00 | 85.71 | 0.56 |
| BOR | 68.67 | 7.42 | 23.91 | 4.26 | 2.13 | 23.40 | 70.21 | 0.08 |
| CON | 76.33 | 8.36 | 15.32 | 22.64 | 6.60 | 10.38 | 60.38 | 0.16 |
| MAC | 43.43 | 10.13 | 46.44 | 0.00 | 3.23 | 4.03 | 92.74 | 0.39 |
| MED | 72.60 | 16.78 | 10.63 | 9.82 | 11.90 | 4.17 | 74.11 | 0.25 |
| PAN | 69.68 | 4.78 | 25.53 | 15.79 | 10.53 | 13.16 | 60.53 | 0.20 |
| STE | 29.74 | 9.02 | 61.24 | 0.00 | 8.33 | 16.67 | 75.00 | 0.21 |


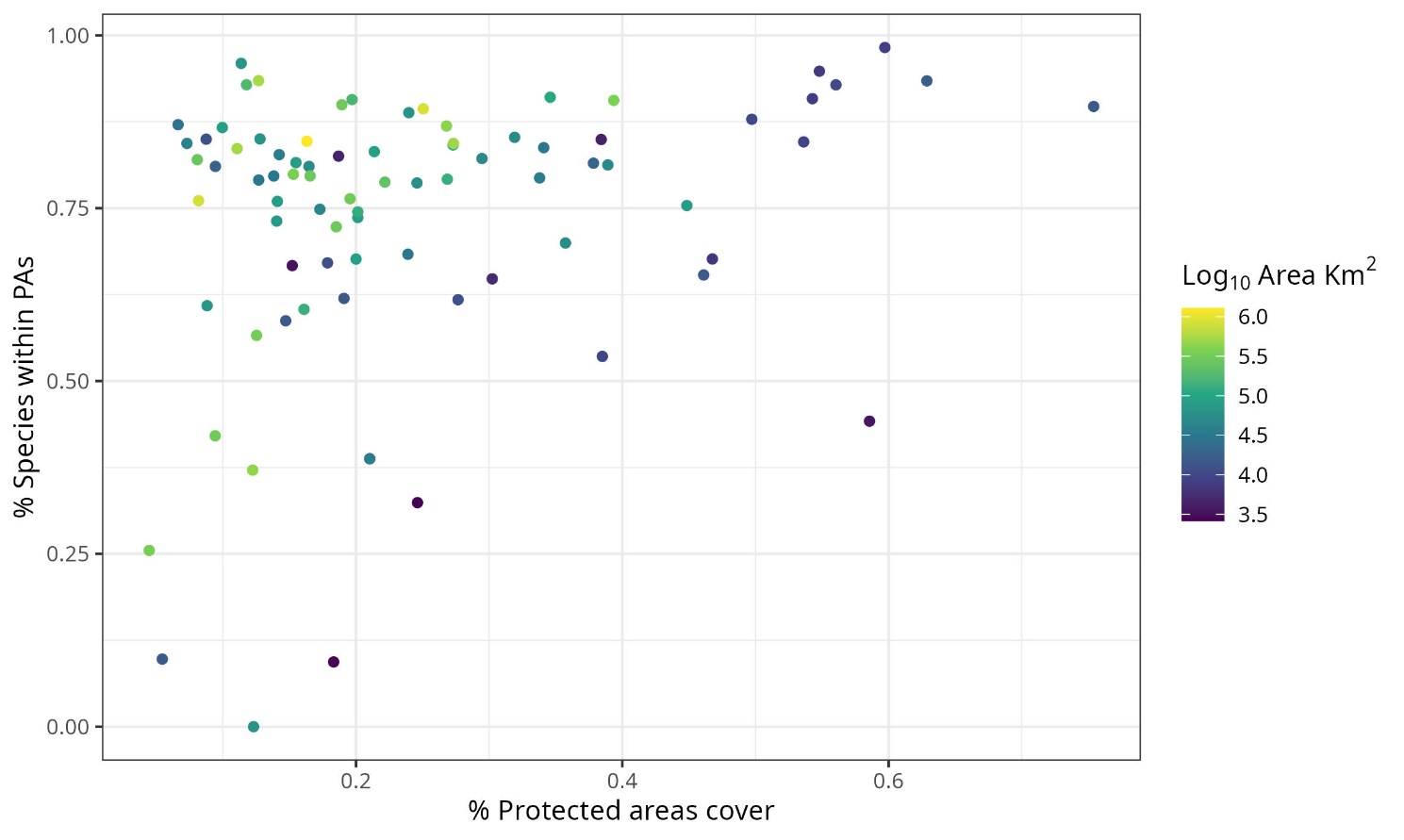
**Appendix S8.** Percentage (%) of native species found within of the Natura 2000 network versus the percentage of land surface covered by the N2K network for each EU country, biogeographical region and their combination of country and biogeographical region of the EU.


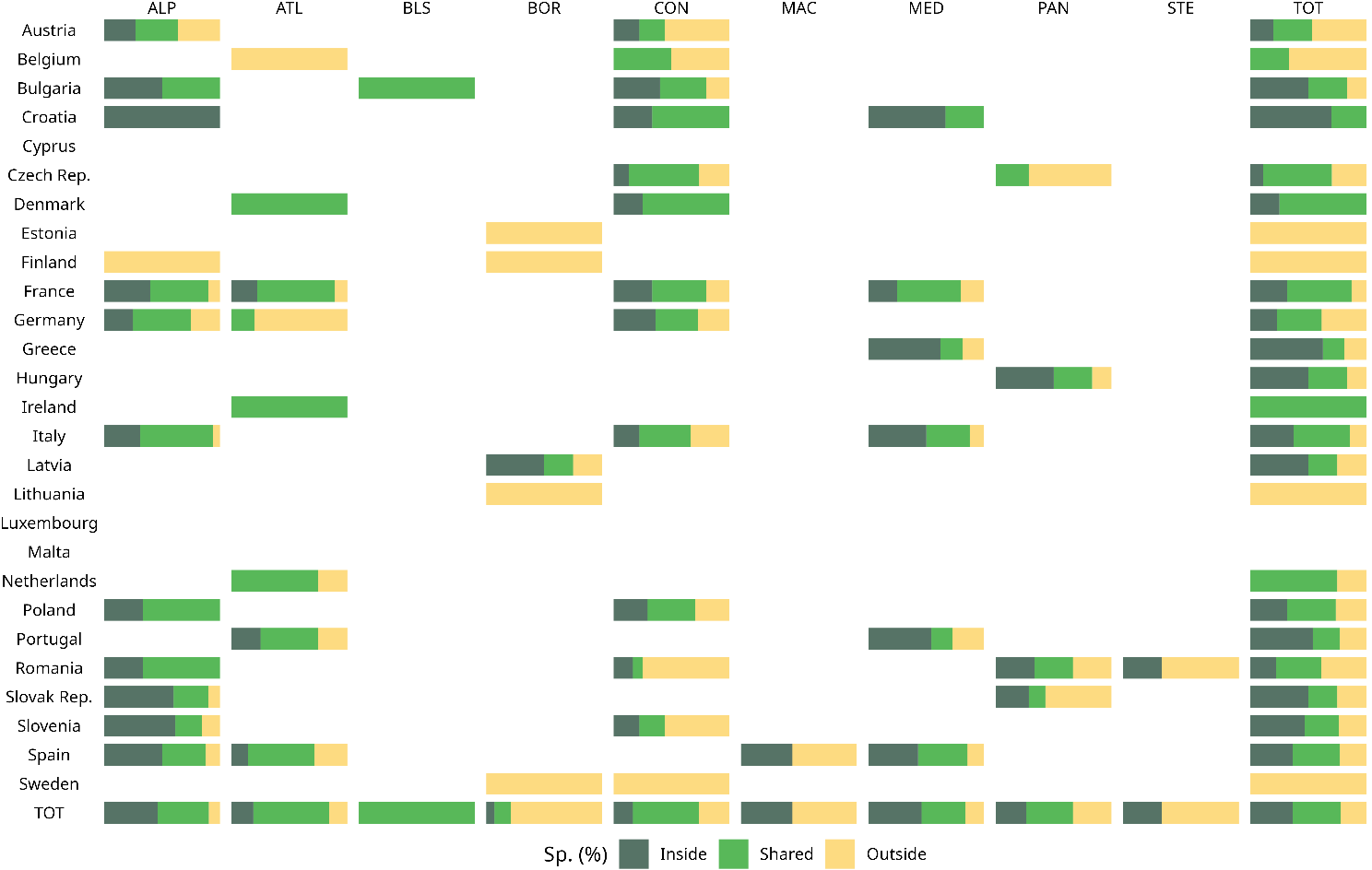
**Appendix S9.** Percentage (%) of priority species found exclusively within, exclusively outside, and shared between areas inside and outside the Natura 2000 network compared to the total reported by the EEA for each combination of country and biogeographical region of the EU. Abbreviations: TOT - Total. See Figure 1 for additional abbreviations.
